# Quality indicators for knee and hip osteoarthritis care: a systematic review

**DOI:** 10.1136/rmdopen-2021-001590

**Published:** 2021-05-26

**Authors:** Ilgin G Arslan, Rianne M Rozendaal, Marienke van Middelkoop, Saskia A G Stitzinger, Maarten-Paul Van de Kerkhove, Vincent M I Voorbrood, Patrick J E Bindels, Sita M A Bierma-Zeinstra, Dieuwke Schiphof

**Affiliations:** 1 General Practice, Erasmus MC University Medical Center, Rotterdam, The Netherlands; 2 General Practice Pallion, Hulst, The Netherlands; 3 Orthopaedics ZorgSaam Zeeuws-Vlaanderen, Terneuzen, The Netherlands; 4 Department of Orthopaedics, Erasmus MC University Medical Center, Rotterdam, The Netherlands

**Keywords:** quality indicators, knee osteoarthritis, osteoarthritis, health care

## Abstract

To provide an overview of quality indicators (QIs) for knee and hip osteoarthritis (KHOA) care and to highlight differences in healthcare settings. A database search was conducted in MEDLINE (PubMed), EMBASE, CINAHL, Web of Science, Cochrane CENTRAL and Google Scholar, OpenGrey and Prospective Trial Register, up to March 2020. Studies developing or adapting existing QI(s) for patients with osteoarthritis were eligible for inclusion. Included studies were categorised into healthcare settings. QIs from included studies were categorised into structure, process and outcome of care. Within these categories, QIs were grouped into themes (eg, physical therapy). A narrative synthesis was used to describe differences and similarities between healthcare settings. We included 20 studies with a total of 196 QIs mostly related to the process of care in different healthcare settings. Few studies included patients’ perspectives. Rigorous methods for evidence synthesis to develop QIs were rarely used. Narrative analysis showed differences in QIs between healthcare settings with regard to exercise therapy, weight counselling, referral to laboratory tests and ‘do not do’ QIs. Differences within the same healthcare setting were identified on radiographic assessment. The heterogeneity in QIs emphasise the necessity to carefully select QIs for KHOA depending on the healthcare setting. This review provides an overview of QIs outlined to their healthcare settings to support healthcare providers and policy makers in selecting the contextually appropriate QIs to validly monitor the quality of KHOA care. We strongly recommend to review QIs against the most recent guidelines before implementing them into practice.

**Key messages**

**What is already known about this subject?**

- Although the evidence-based recommendations for the management of knee and hip osteoarthritis (KHOA) are internationally similar, clinical practice is context-dependent and therefore varies between countries.
- Previous research has shown that quality indicators (QIs) cannot simply be transferred between countries, due to structural and cultural differences of healthcare systems.

**What does this study add?**

- This review provides an overview of QIs for KHOA care showing considerable differences between QIs depending on their healthcare settings.

**How might this impact on clinical practice or further developments?**

- This overview will support healthcare providers and policy makers in selecting the contextually appropriate QIs to validly monitor the quality of care, but we strongly recommend to review the QIs against the most recent guidelines before implementing them into practice.

## Background

Osteoarthritis (OA) is one of the leading musculoskeletal causes of global disability, mainly affecting the knees and the hips.^1^ The prevalence has increased worldwide with 32% between 2005 and 2015 and is expected to increase even more with the ageing of the population and the rising obesity rate. This will become a challenge for the health systems globally.^2–4^

Despite the presence of numerous consistent guidelines for the management of knee and hip OA (KHOA),^5–9^ clinical practice shows a low consistency with following these recommendations leading to suboptimal care.^10 11^ Therefore, routinely monitoring of feedback on quality of care has been made high priority.^12^ Quality indicators (QIs) are measurable elements that can be used to assess the quality of care. These QIs can be related to the characteristics of material and human resources of the healthcare (ie, the structures), activities undertaken in the delivered healthcare (ie, the process) and the changes in health status resulting from the delivered healthcare (ie, the outcomes).^13–15^

Although the evidence-based recommendations for the management of KHOA are internationally similar, clinical practice is context-dependent and therefore varies between countries. In the Netherlands, Scandinavian countries and the UK, the content of KHOA treatment depends on the healthcare setting. Non-surgical management of KHOA is largely provided in primary care. For patients who do not respond successfully to this approach, a referral to secondary care for surgical management is indicated.^5 16^ This distinction in healthcare settings is less pronounced in other countries such as the USA, where the first point of contact and access to orthopaedic care strongly depend on patients’ health insurance status.^17 18^ Previous research has shown that QIs cannot simply be transferred between countries, due to structural and cultural differences of healthcare systems.^19^ This has led to a variety of QIs for OA care.

Several systematic reviews have focused on QIs for OA in primary care.^20 21^ However, an overview of QIs that take into account the differences in healthcare settings and countries is lacking. Such an overview will support healthcare providers and policy makers in selecting the contextually appropriate QIs. This will enable them to validly monitor and provide feedback on the quality of care.

Therefore, the aim of this systematic review was to provide an up-to-date overview of QIs for KHOA in which we outline the healthcare settings and countries for which the QIs have been developed or adapted.

## Methods

This systematic review was conducted and reported in line with the Preferred Reporting Items for Systematic Reviews and Meta-Analyses statement.^22^ A protocol for conducting this systematic review was developed a priori and is available on request.

### Search methods for identification of studies

An electronic database search was conducted by a trained medical librarian up to March 2020, using MEDLINE (PubMed), EMBASE, CINAHL, Web of Science, Cochrane CENTRAL and Google Scholar databases. For unpublished and ongoing studies, a similar search was conducted in OpenGrey and the Prospective Trial Register database. A range of search terms related to OA (eg, osteoarthrit*, hip, knee) combined with indicator terms (eg, quality*, indicator, process, structure) were used to identify studies. Full details of the search strategy are provided in online supplemental file 1. The electronic database search involved no restrictions on healthcare setting, country, language, study design and publication status. Reference lists of studies were manually searched recursively until no additional eligible publications were identified.

10.1136/rmdopen-2021-001590.supp1 Supplementary data

### Criteria for considering studies for this review

#### Type of studies

Studies about the development of QI(s) and adaptation of existing QI(s) for another context were included (eg, cross-sectional studies, literature reviews and Delphi studies). Reviews that contained QIs which were already included from other studies were excluded, as were conference abstracts and studies written in languages other than English, Scandinavian, Dutch, Turkish and German. Studies published before January 2000 were excluded, since they may contain QIs that are more likely to be outdated and may therefore include treatment modalities that are no longer recommended. Studies focusing on patients with OA and other diseases (eg, rheumatoid arthritis (RA)) were included if QIs about OA were presented separately.

#### Type of QIs

QIs for OA care, either specifically in the knees and hips or OA not related to specific sites, were extracted from the studies. QIs that measure postsurgical healthcare (eg, after joint replacement) were excluded. Various types of individuals (eg, patients, healthcare providers or healthcare managers) could be involved in the adaptation or development process of the QIs, resulting in QIs from various perspectives of stakeholders.^13 23^ QIs from all types of perspectives were included in this review.

### Data collection and analysis

All titles and abstracts were double and independently screened for their relevance (IGA plus DS or RR). Full-texts of potentially eligible studies were gathered and screened again by double independent review to check for their relevance (IGA plus DS or RR). Data from the included studies were extracted into a pretested data extraction form by one reviewer (IGA) and checked by another reviewer (DS or RR). The following data were extracted: general information about the study, healthcare setting, country, target population, involved joints (eg, knee OA, hip OA or any OA), perspective of QI(s), information of testing and implementation of the QI(s) if this was done in the study and the full QI(s). Furthermore, methods of evidence synthesis and consensus method were extracted. An evidence synthesis using a systematic review and consensus method using a RAND Appropriateness Method or a Delphi method were considered as the most rigorous methods.^24 25^ Possible conflict of interest due to funding and non-adherence to the study protocol were extracted and considered as a source of bias. Disagreements in data collection were resolved by consensus and if necessary, by the third reviewer. The extracted QIs were then categorised into three categories according to Donabedian, which conceptualises quality of care through the structures, processes and outcomes of care (online supplemental figure 1).^13 14^ Structure QIs refer to attributes of material and human resources used for providing care (eg, percentage of specialists among all doctors). Process QIs reflect the activities undertaken in the delivered care (eg, percentage of patients who are offered exercise therapy among all patients). Outcome QIs refer to changes in health status as a result of the delivered care (eg, percentage of patients with functional improvement among all patients). Within these three categories, QIs were grouped in themes (eg, QIs for medication, QIs for weight loss, etc). For the purpose of narrative analysis, we categorised studies into healthcare settings, for example, primary care setting or secondary care setting. Within each category and theme, differences and similarities between the healthcare settings were analysed and summarised. Authors of studies (n=3, response rate=100%) were contacted for additional information for the data collection and analysis.

10.1136/rmdopen-2021-001590.supp2 Supplementary data

## Results

### Results of the search

**Figure 1:**
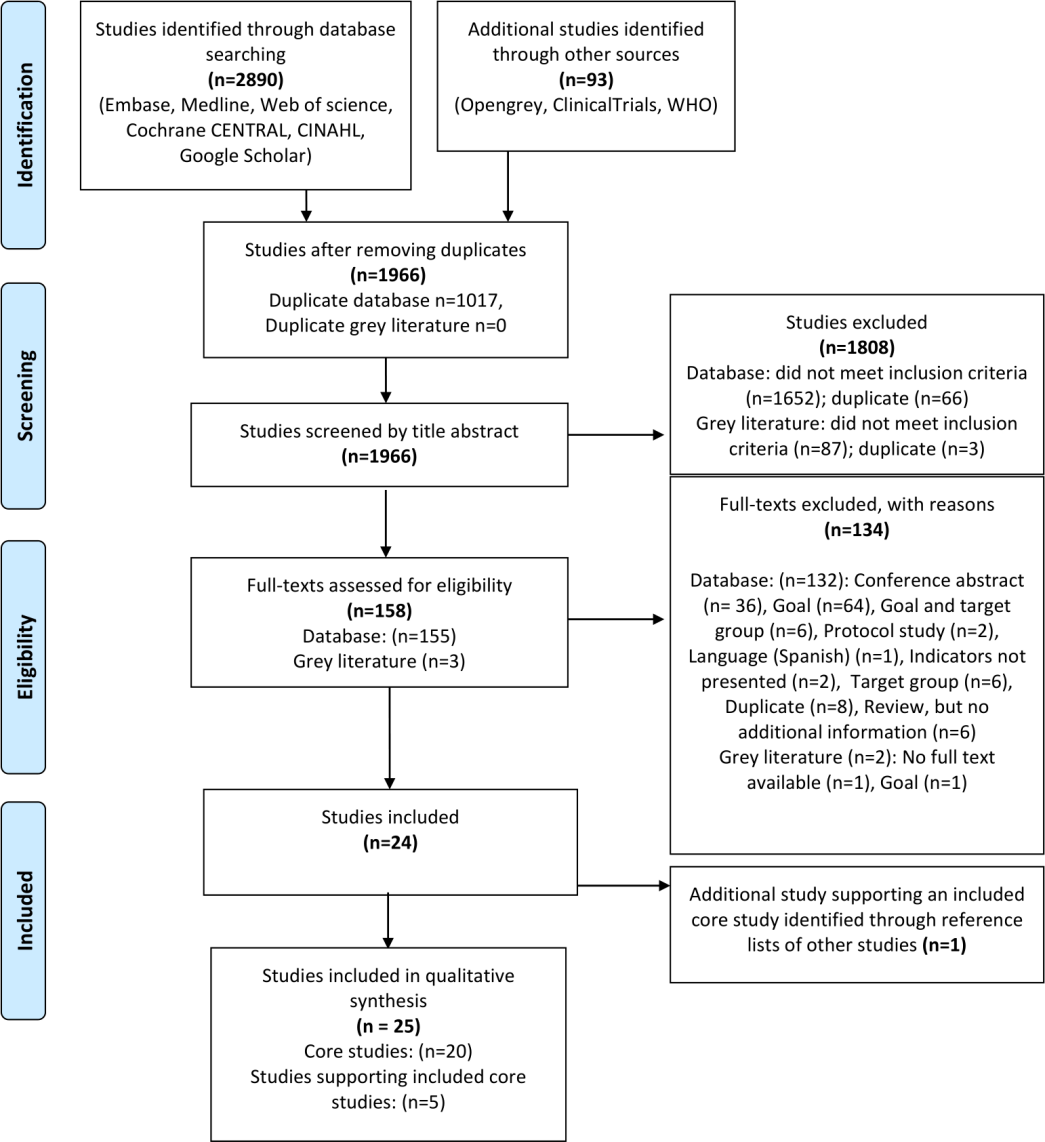
Flow chart for the selection of studies. From: Moher *et al*.^61^

The search strategy identified 1966 studies, after removing duplicates (figure 1). After screening on title and abstract, 1808 studies were excluded. The remaining 158 studies were screened on full-texts, of which 24 studies were included. One additional study^26^ was identified through reference lists of included studies. The main reasons for exclusion on full-text are listed in online supplemental file 2. Of the 25 included studies, 5 studies^26–30^ described the methods of other already included studies (ie, core studies) in detail. We did not exclude these studies, but used them as supporting studies for data extraction and analyses, as they contained additional information not reported in the core studies.

10.1136/rmdopen-2021-001590.supp3 Supplementary data

### Characteristics of the studies included

#### Methods of development of QI(s) or adaptation of existing QI(s)

The characteristics of the studies included are summarised in table 1 and more detailed in online supplemental file 3. Only 5 out of 20 studies (25%) included an evidence synthesis for the QIs using a systematic review.^31–35^ Consensus on QIs during the development phase was mostly done using a (modified) RAND Appropriateness Method^19 31–34 36–40^ or Delphi method.^41 42^ The remaining studies used less rigorous methods^35 43–46^ or did not specify the methods.^47 48^ Four studies^36 44–46^ tested the reliability of the QIs. Eleven studies^19 36 37 43 44 46–48^ evaluated the feasibility of QIs in practice and three studies^34 38 39^ through judgement by an expert panel. Although not every study reported information on conflict of interest, the reviewers judged most of the studies unlikely to have conflict of interest. No study protocols of the studies included were available, hence no judgement about adherence to the protocol could be made. All studies included QIs in the process of care category. Three studies^34 35 43^ included QIs in the process and outcome category, and only one study^34^ in all three categories. Information on healthcare perspectives of the QIs (ie, types of individuals involved with the development/adaptation process of the QIs) was often not reported. Studies that reported the healthcare perspectives for developing QIs or adapting existing QIs mostly involved the perspectives of healthcare professionals^19 34 37–39 44 49^ and researchers,^32 43 46^ and in a few cases the perspectives of patients^34 35^ and healthcare organisations.^34^ QIs were often developed to measure the quality of care with data from paper or electronic records. Some studies^35 38^ developed QIs to measure the quality of care with data from patient or physiotherapist-reported forms^43 44 46^ or a mixture of patients or proxy interviews and medical records.^31–33^

10.1136/rmdopen-2021-001590.supp4 Supplementary data

**Table 1:** Characteristics of the studies included (n=20 studies)

Study	Perspective of quality of care	Level of care	Proposed method of QI derivation	Evidence synthesis	Consensus method	Testing/implementation	
Asch *et al*^36^	NR	Process	Medical records	Literature review, not specified to be systematic	RAND approach/modified Delphi method	Implemented in 12 Veterans Health Administration care systems and 12 US communities. Average reliability: presence of a condition (κ=0.83), indicator eligibility (κ=0.76) and indicator scoring (κ=0.80)	
Barber *et al*^34^	Healthcare professionals, organisational and patients	Structure, process and outcome	NR	Integrative review including an update of an earlier conducted systematic review	Modified RAND-UCLA appropriateness method	Feasibility assessed by the expert panel during the Delphi rounds	
Blackburn *et al*^35^	Patients	Process and outcome	Patient-reported questionnaire	Used an earlier published systematic review^21^	Four discussion groups with the research team	Not tested/implemented	
Broadbent *et al*^48^	NR	Process	Medical records	Used QIs of a published study,^6^ and of the National Primary Care Research and Development Centre	NR	QIs implemented in 18 general practices in the UK	
Doubova and Perez-Cuevas^37^	Healthcare professional	Process	Electronic health records	Literature review, not specified to be systematic	Modified RAND/UCLA appropriateness method	QIs implemented cross-sectional in four family medicine clinics in Mexico City	
Grypdonck *et al*^49^	Healthcare professional	Process	NR	Literature review, not specified to be systematic	RAND-modified Delphi method	Not tested/implemented	
Hardcastle *et al*^38^	Healthcare professional	Process	Patient interview surveys	Used QIs of an earlier published study^40^	Modified RAND/UCLA appropriateness method	Feasibility of the survey use assessed by an expert panel of clinicians	
Jansen *et al*^43^	Researchers	Process and outcome	Physiotherapist self-reported recording forms	QIs were derived from the Dutch physiotherapy guideline on KHOA	Independent assessment of QIs by two authors	QIs implemented by 27 physical therapists who recorded patient and treatment characteristics of at least five consecutive patients	
MacLean^31^ : ACOVE-1 (supporting article: Shekelle *et al*)^26^	NR	Process	Medical records, administrative data and patient or proxy interview	Systematic review	Modified RAND/UCLA appropriateness method	Not tested/implemented	
MacLean *et al*^33^ : ACOVE-2 (supporting article: Pencharz and MacLean)^29^	NR	Process	Medical records, administrative data and/or patient or proxy interview	Systematic review	Modified RAND/UCLA appropriateness method	Not tested/implemented	
MacLean *et al*: ACOVE-3^32^	Healthcare professionals	Process	Medical records, administrative data and/or patient or proxy interview	Systematic review	Modified RAND/UCLA appropriateness method	Not tested/implemented	
Marshall *et al*^19^	Healthcare professionals	Process	Electronic and paper records from the general practice	Literature review, not specified to be systematic	RAND/UCLA appropriateness method	Field-testing on 1600 randomly selected patient records in 16 general practices	
Moore^39^	Healthcare professionals	Process	Medical records	Literature review, not specified to be systematic	RAND/UCLA appropriateness method	Feasibility of the QIs assessed by the expert panel	
Østerås *et al*^46^ (supporting article: Østerås *et al*^30^	Researchers	Process	Patient self-reported questionnaire	Literature review, not specified to be systematic	Critical judgement by researchers and national and international colleagues that used the questionnaire in different settings	Feasibility of QIs assessed during 2010–2014 in a Norwegian OA cohort (V1). Revised version, the OA-QI v2, was then pilot-tested by 11 of the members in the Patient Research Partner Panel at Diakonhjemmet Hospital. Test–retest к=0.38–0.85, exact agreement from 69% to 92%. The ICC for all 16 items was 0.89	
Peter *et al*^44^	Healthcare providers	Process	PT self-reported online questionnaire	QIs derived from the Dutch physiotherapy guideline on KHOA	Rating of recommendations of guideline by an expert panel of PTs	Pilot-tested by 15 PTs and three experts. Consecutively, pilot test is done among expert (n=51) and PTs (n=192) in the Netherlands. Test–retest reliability: ICC=0.89	
Saliba *et al*^42^	NR	Process	Not reported	Adapted ACOVE-1 set for nursing homes	Modified Delphi process	Not tested/implemented	
Smith *et al*^41^	NR	Process	Not reported	Adapted ACOVE-1 set for home-based care	Modified Delphi process	Not tested/implemented	
Steel *et al*^40^	NR	Process	Medical records	Adapted ACOVE-1 set for UK healthcare system	Modified RAND/UCLA appropriateness method	Not tested/implemented	
Vandenberghe *et al*^47^	NR	Process	Paper registration sheets or electronic patient records	NR	NR	QIs implemented cross-sectionally in the general practices in Belgium and compared between a pooled and restricted database	
Wierenga *et al*^45^	NR	Process	Medical records and a hospital information system	Adapted ACOVE-1 set for in-hospital pharmaceutical care	Expert panel review methods	Feasibility assessment with ten elderly patients. Reliability tested with ten randomly selected patients; к=0.88 (95% CI 0.75 to 1.00); ICC=0.80 (95% CI 0.63 to 0.90)	

More information on the data extraction and quality assessment for each study is provided in online supplemental file 3.

ACOVE, Assessing Care of Vulnerable Elders; ICC, intraclass correlation coefficient; KHOA, knee and hip osteoarthritis; NR, not reported; OA, osteoarthritis; PT, physiotherapist; RA, rheumatoid arthritis.

#### Healthcare settings

Studies were categorised into five healthcare settings: primary care (n=10), secondary care (n=3), the entire spectrum of disciplines (n=8) and centralised intake care (n=1) (table 2). Nine studies^19 35 37 40 41 43 44 47 48^ developed QIs for primary care, mainly on healthcare in general practice and physiotherapy care. Three studies developed QIs for secondary care in the USA,^42^ the Netherlands^45^ and the UK.^40^ We categorised eight studies as targeting the entire spectrum of disciplines since they did not focus on a specific healthcare setting. Five of those^31–33 36 39^ developed QIs for the healthcare system in the USA, of which three^31–33^ developed the Assessing Care of Vulnerable Elders QI set. Of the remaining studies targeting the entire spectrum of disciplines, one study^49^ was conducted in Belgium, one study^38^ focused on UK private households and one study^46^ on the Norwegian healthcare system. Another study^34^ developed QIs for a relatively new and exceptional system in Canada; the centralised intake care. This system pools patients into a single queue, assesses the nature and urgency of referral and prioritises the access to care based on this assessment.

**Table 2:** Included studies (n=20) categorised according to their healthcare setting

Study	Target population	Country
*Primary care*		
Blackburn *et al*^35^	Patients with OA in primary care setting	UK
Broadbent *et al*^48^	Patients with OA in general practice	UK
Doubova and Perez-Cuevas^37^	Patients with KHOA aged ≥19 in family medicine	Mexico
Jansen *et al*^43^	Patients with KHOA in PT care	The Netherlands
Marshall *et al*^19^	Patients with OA in general practice	UK
Peter *et al*^44^	Patients with KHOA in PT care	The Netherlands
Smith *et al*^41^	Housebound elderly patients in home-based primary care	USA
Steel et al**^40^	People aged 65 and with OA in primary and secondary care	UK
Vandenberghe *et al*^47^	Patients with OA of aged ≥60 in general practice	Belgium
*Secondary care*		
Saliba *et al*^42^	Institutionalised vulnerable elderly with OA in nursing homes	USA
Steel et al**^40^	People aged 65 and with OA in primary and secondary care	UK
Wierenga *et al*^45^	Elderly hospitalised patients with OA from in-hospital pharmaceutical care	The Netherlands
*The entire spectrum of disciplines*		
Asch *et al*^36^	Patients of outpatient and inpatient care for acute and chronic conditions and preventive care (including OA)	USA
Grypdonck *et al*^49^	Patients with knee OA across the entire spectrum of disciplines	No country specified
Hardcastle *et al*^38^	People with OA aged ≥50 living in private households	UK
MacLean^31^	Vulnerable elderly with OA	USA
MacLean *et al*^33^	Patients with OA	USA
MacLean *et al*^32^	Vulnerable elderly with OA	USA
Moore^39^	Patients with OA	USA
Østerås *et al*^46^	Patients with OA	Norway
*Centralised intake care system*		
Barber *et al*^34^	Patients with RA and/or OA in centralised intake care system	Canada

*Steel *et al*^40^ is listed twice in the table, as it focuses on ‘primary care’ and ‘secondary care’.

KHOA, knee and hip osteoarthritis; OA, osteoarthritis; PT, physiotherapy; RA, rheumatoid arthritis.

### Narrative synthesis

A total of 196 QIs were derived from the included studies. See online supplemental table 4 for a detailed description of the QIs with the actual wordings as stated in the original studies, grouped by category and theme.

10.1136/rmdopen-2021-001590.supp5 Supplementary data

#### QIs about the structure of care

With respect to the structure of care, one study^34^ developed three QIs for centralised intake care in Canada concerning the completion of appointments as scheduled, number of specialist providers participating in centralised intake and clinic capacity of the OA teams (table 3).

**Table 3:** Quality indicators on structure of care (n=3)

Theme	Subtheme (number of QIs)	Healthcare setting and country
Musculoskeletal appointments	Musculoskeletal appointments completed as scheduled (n=1)	Centralised intake care system in Canada^34^
Healthcare providers involved	Specialist providers participating in centralised intake (n=1)	Centralised intake care system in Canada^34^
Estimation of clinic capacity	Ratio of patient flow to estimated clinic capacity of OA teams participating in centralised intake (n=1)	Centralised intake care system in Canada^34^

OA, osteoarthritis; QI, quality indicator.

#### QIs about the process of care

Regarding the process of care, we identified QIs on 10 different themes (table 4).

**Table 4:** Quality indicators on process of care (n=182)

Theme	Subtheme (number of QIs)	Healthcare setting and country
History taking and examination (n=32)	Regular assessment of functional status and pain (n=9)	Primary care in the UK, USA and Norway.^40 41 46 48^ Secondary care in the UK and USA.^40 42^ The entire spectrum of disciplines in the USA.^31–33^
	Assessment for assistive devices, appliances and aids (n=6)	The entire spectrum of disciplines in the USA and Norway.^32 33 46^
	Radiographic assessment (n=3)	The entire spectrum of disciplines in the USA and one study with unspecified country.^33 39 49^
	Diagnostic aspiration (n=4)	Primary care in the USA.^41^ Secondary care in the USA.^42^ The entire spectrum of disciplines in the USA and one study with unspecified country.^31 49^
	Inventory of health-related problems (n=4)	Primary care; PT care in the Netherlands.^44^ The entire spectrum of disciplines in Norway.^46^
	Examination of joint before drug treatment (n=2)	Secondary care in the USA.^42^ The entire spectrum of disciplines in the USA.^33^
	Health assessment for evaluation of treatment (n=4)	Primary care; PT care in the Netherlands^44^
Education and information (n=22)	Information and advice concerning pathology of OA, lifestyle and physical activity formulated in detail (n=9)	Primary care; PT care in the Netherlands.^44^ The entire spectrum of disciplines in Norway.^46^
	Information concerning joint protection and the use of aids (n=1)	Primary care; PT care in the Netherlands^44^
	Advise about medication (n=1)	Primary care in the UK.^35^
	Information concerning pathology of OA, treatment and self-management formulated in general (n=10)	Primary care in the UK.^35 40 48^ Secondary care in the UK.^40^ The entire spectrum of disciplines in the USA, Norway and one study with unspecified country.^31 33 38 46 49^
	Information regarding resources and tools while waiting for an appointment (n=1)	Centralised intake care system in Canada^34^
Exercise therapy (n=25)	Exercise therapy, recommendation/prescription for activities, of strengthening, aerobic exercises and functional exercises body functions and walking exercises (n=4)	Primary care in the UK and PT care in the Netherlands^35 44^
	Recommendation/prescription (n=15)	Primary care in the USA, UK, Mexico and PT care in the Netherlands.^41^,^37 40 44^ Secondary care in the USA and UK.^40 42^ The entire spectrum of disciplines in the USA, UK and one study with unspecified country.^31–33 38 49^
	Recommendation of exercise therapy formulated in general (n=2)	The entire spectrum of disciplines in the USA^36 39^
	Combining exercise therapy with education/self-management interventions, frequency and evaluation, and tailoring exercise therapy to patients’ goals (n=4)	The entire spectrum of disciplines, country not specified^49^
Weight counselling (n=7)	Advice about body weight and joint pain (n=7)	Primary care in the UK and Mexico.^35 37^ The entire spectrum of disciplines in the USA, Norway and one study with unspecified country.^33 39 46 49^
‘Do not do’ QIs (n=3)	No massage therapy, no prescription of a brace and no physical modalities other than TENS (n=3)	Primary care; PT care in the Netherlands.^43^ The entire spectrum of disciplines; country not specified.^49^
Pharmacological treatment (n=51)	Paracetamol as first-line pharmacological therapy (n=16)	Primary care in the USA, UK, Belgium and Mexico.^19 37 40 41 47 48^ Secondary care in the Netherlands, UK and USA.^40 42 45^ The entire spectrum of disciplines in the USA, UK, Norway and one study with unspecified country.^31–33 36 38 39 46 49^
	Trial of maximum-dose acetaminophen before changing from acetaminophen to different oral agent (n=7)	Primary care in the UK and USA.^40 41 48^ Secondary care in the USA, UK and the Netherlands.^40 42 45^ The entire spectrum of disciplines in the USA.^31 33^
	Prescription of NSAIDs and concomitant with either misoprostol or proton-pump inhibitor (n=15)	Primary care in the UK, Belgium and Mexico.^19 37 47 48^ The entire spectrum of discipline in the USA and one study with unspecified country.^31 32 49^
	Informing patients about risks of medication use and screening for side effects (n=8)	Primary care in the USA and UK.^41 48^ The entire spectrum of disciplines in the USA and Norway.^31 32 46^
	Injection (n=1)	The entire spectrum of disciplines in Norway^46^
	No medication use of several drug types, that is, chondroitin and glucosamine-chondroitin and strong pain killers such as opioids (n=4)	Primary care in Belgium.^47^ The entire spectrum of disciplines in the USA and Norway.^46 49^
Referrals (n=26)	Exercise therapy/programmes/activities (n=5)	Primary care in the UK.^35^ The entire spectrum of disciplines in Norway and one study with unspecified country.^46 49^
	Weight loss services (n=3)	Primary care in the UK.^35^ The entire spectrum of disciplines in the USA and Norway.^33 46^
	Orthopaedic surgeon (n=8)	Primary care in the UK.^19 40 48^ Secondary care in the UK.^40^ The entire spectrum of disciplines in the USA and Norway.^31–33 38 46^
	Laboratory tests (n=1)	Primary care in Mexico^37^
	Centralised intake care specific QIs, for example, time from referral to appointment (n=9)	Centralised intake care system in Canada^34^
Indications for surgical treatment (n=4)	Indication for knee replacement (n=1)	The entire spectrum of disciplines; country not specified^49^
	Unicompartmental knee replacement (n=1)	The entire spectrum of disciplines; country not specified^49^
	No arthroscopic interventions of the knee (n=1)	The entire spectrum of disciplines; country not specified^49^
	Operating room time (n=1)	Centralised intake care system in Canada^34^
Documentation (n=6)	Symptoms, limitations in daily activities, systemic or inflammatory disease, physical examination and use and effectiveness of treatment (n=3)	The entire spectrum of disciplines in the USA^36 39^
	Presence of systemic or inflammatory disease, and joint trauma or surgery (n=1)	The entire spectrum of disciplines in the USA^39^
	Problem areas and patient profile (n=2)	Primary care; PT care in the Netherlands^43^
Follow-up, treatment frequency, duration and aftercare (n=6)	Follow-up review (n=2)	The entire spectrum of disciplines in the USA.^39^ Centralised intake care system in Canada.^34^
	Treatment frequency, number of sessions and duration of treatment episode (n=3)	Primary care; PT care in the Netherlands^43^
	Aftercare (eg, home exercise programme) (n=1)	Primary care; PT care in the Netherlands^43^

NSAID, non-steroidal anti-inflammatory drug; OA, osteoarthritis; PT, physiotherapy; QI, quality indicator; TENS, transcutaneous electrical nerve stimulation.

##### History taking and examination (n=32 QIs)

QIs on assessment of functional status and level of pain were most common and focused on all healthcare settings, except for centralised intake care. QIs on assessment for assistive devices, appliances and aids, and radiographic assessment also focused on the entire spectrum of disciplines, except for centralised intake care. Differences were seen in the indication for radiographic assessment; from offering a radiography to patients with incident hip OA to only offering a radiograph to patients with worsening complaints or patients who seem resistant to conservative treatment. QIs on the diagnostic aspiration of the joint and examination of joint before drug use were less common and focused on the US only. QIs relating to history taking and health assessment to evaluate the given treatment were mainly described for (physiotherapy) primary care settings in Europe.

##### Education and information (n=22 QIs)

QIs on this theme related to information on the pathology of OA, treatment options and self-management and were similar between countries. Most QIs on this theme were developed for primary care (physiotherapy) in the Netherlands, but least for secondary care and healthcare in the US.

##### Exercise therapy (n=25 QIs)

QIs regarding exercise therapy were mostly developed for primary care on recommending and prescribing physiotherapy or specific exercises and were similar between countries. Three QIs focusing on the entire spectrum of disciplines were found regarding the frequency and regular evaluations of exercise therapy sessions, and regarding tailoring exercise therapy to patients goals.

##### Weight counselling (n=7)

QIs for advice on weight loss were developed for primary care and the entire spectrum of disciplines. Body mass index (BMI) threshold and frequency for advising patients to lose weight differed between QIs for the entire spectrum of disciplines in the USA and QIs for family medicine in Mexico (>25 kg/m^2^ vs >27 kg/m^2^, and at least once in 2 years vs annually).

##### ‘Do not do’ QIs (n=3)

Two QIs for primary care (physiotherapy) in the Netherlands focused on recommending against massage and physical modalities other than Transcutaneous Electrical Nerve Stimulation. One QI for the entire spectrum of disciplines focused on not prescribing a brace for people with knee OA, except for patients with unicompartmental knee OA with axial deviation.

##### Pharmacological treatment (n=51)

Most of the pharmacological treatment QIs were developed for primary care. These QIs were consistent in their content and covered: (1) the use of paracetamol as first-line pharmacological therapy, (2) prescribing a trial of maximum-dose paracetamol before changing to a different oral agent, (3) non-steroidal anti-inflammatory drugs (NSAIDs) prescription, (4) NSAID prescription concomitant with either misoprostol or proton-pump inhibitor and (5) informing/screening patients about the risks of medication use. One additional QIs for the entire spectrum of disciplines in Norway focused on the indication of injections.^46^ Four QIs focused on not using several drug types, mainly focusing on primary care. One QI covered not using strong opioids and one QI not using chondroitin and glucosamine-chondroitin.^49^ A Norwegian study^46^ formulated a QI that offering stronger pain killers in OA patients (eg, co-proxamol, co-dydramol, tramadol, co-codamol, dihydrocodeine, codeine) in case of no sufficient pain relief by paracetamol reflects better quality of care.

##### Referrals (n=26)

Four QIs were found regarding the referral of patients to exercise therapy/programmes/activities in all studies included in this study, except in studies focusing on Mexico and the USA. From the three QIs that focused on referral for weight loss services, only one^33^ defined a specific threshold for BMI for the referral to weight loss services (US healthcare). QIs regarding the referral to an orthopaedic surgeon when patients do not respond sufficiently to non-surgical therapy were similar in all studies. There was only one QI for family medicine in Mexico regarding referral to laboratory test to detect possible adverse events.^37^ The remaining QIs (n=6) focused on centralised intake care in Canada,^34^ for example, regarding the agreement of centralised intake suspected diagnosis of severe OA cases versus confirmed diagnosis of severe OA.

##### Indication of surgery (n=4)

Only two studies developed QIs on the indication for surgical treatment. One study for the entire spectrum of disciplines^49^ developed QIs for indications for different types of surgical treatments for knee OA (ie, joint replacement and arthroscopic interventions) and one study^34^ for centralised intake care system in Canada regarding operating room time. QIs regarding indications for surgical treatment for hip OA are lacking. Remarkably, studies that focused on secondary care^40 45^ did not develop QIs for the indications for surgical treatment.

##### Documentation (n=6)

Six QIs were found on documentation of information on measures from physical examination for the entire spectrum of disciplines in the USA^36 39^ and on patients’ characteristics for primary care (physiotherapy) in the Netherlands.^43^

##### Treatment frequency, duration, follow-up and aftercare (n=6)

Although not all QIs on this theme defined a specific threshold, three QIs for primary care (physiotherapy) in the Netherlands and one for the entire spectrum of disciplines in the US healthcare specified a threshold for treatment frequency (<12 consultations), duration (<6 weeks) and follow-up (every 6 weeks). The study on primary care (physiotherapy) in the Netherlands^43^ was also the only one that developed a QI for aftercare, for example, regarding home exercise programmes.

#### QIs at outcome level of care

QIs at outcome level of care included experiences and satisfaction with healthcare (n=6), pain and functional capacity (n=3) and achievement of treatment goals (n=1) (table 5). The QIs on satisfaction and experiences of healthcare providers and patients were mostly developed for centralised intake care in Canada. The QIs on the other themes were developed for primary care (physiotherapy) in the Netherlands.^43^ For most of the QIs on outcome level of care, the threshold reflecting high or low quality of care was not specified (eg, QI: ‘the extent to which the treatment goals were achieved’^43^).

**Table 5:** Quality indicators on outcome of care (n=11)

Theme	Subtheme (number of QIs)	Healthcare setting and country
Experiences and satisfaction with healthcare (n=6)	Healthcare providers’ and patients’ experiences (n=4)	Centralised intake care system in Canada^34^
	Patients’ satisfaction (n=2)	Primary care in the UK and PT care in the Netherlands^35 43^
Pain and functional capacity (n=4)	Level of pain and functional capacity (n=3)	Primary care; PT care in the Netherlands^43^
Achievement of treatment goals (n=1)	The extent to which the treatment goals were achieved (n=1)	Primary care; PT care in the Netherlands^43^

PT, physiotherapy; QI, quality indicator.

## Discussion

This systematic review provides an overview of 20 studies including a total number of 196 QIs for KHOA care for a variety of healthcare settings. Rigorous methods for evidence synthesis to develop QIs were rarely used in the included studies. Adequate reporting on the perspective of healthcare, the proposed method of measurement (eg, medical records) and threshold of the QIs was lacking. QIs were mainly developed from the perspective of healthcare professionals and researchers, while a patient perspective is limited. Narrative analysis showed that most healthcare settings and countries contain QIs on the following themes with largely similar content: (1) examination of functional status and pain, (2) education and information, (3) exercise therapy, (4) referral to exercise therapy/programmes/activities (5) and pharmacological treatment regarding paracetamol, NSAID and risks of medication use. For example, regarding the use of paracetamol as first-line pharmacological therapy and prescribing a trial of maximum-dose paracetamol before changing to a different oral agent. Some differences in the content of QIs occur due to the healthcare system, that is, QIs about exercise therapy, weight counselling, referral to laboratory tests and ‘do not do’ QIs (mainly described for physiotherapy care in the Netherlands). Nevertheless, differences in the content of QIs occurred within the same healthcare setting with regard to indications for radiographic assessment of the joint.

Studies in the current review included mostly QIs that were related to the process of care. An explanation therefore could be that the studies included developed QIs or adapted existing QIs for quality of care improvement purposes. Process measures offer a roadmap for improving care or list the actions required to eventually improve outcomes for quality improvement purposes. In contrast, outcome measures are mainly developed for public reporting and accountability purposes through feedback on quality of care in order to stimulate quality improvement rather than specific actions to improve the quality of care.^50^ Another explanation might be that outcome measures in OA care mainly focus on reduction in pain and functional improvement. These outcome measures are not easy to capture within daily practice as a process of care. In contrast, for example, blood tests to measure disease activity of RA are captured as a process of care for patients with RA, which makes it easier to evaluate this measure as an outcome of care. However, the low number of QIs on structure of care remains unclear. Most QIs on outcome level were developed in the physiotherapy care in the Netherlands. These QIs are derived from the Dutch KHOA guidelines for physiotherapy with great focus on the outcomes of therapy.

This study identified differences within themes of QIs, which can be explained by differences between healthcare settings and countries. First, QIs for physiotherapy care in the Netherlands strongly focused on inventory of health-related problems, education and information, and exercise therapy. This is likely explained by the fact that the management of KHOA in physiotherapy care focuses on non-surgical and non-pharmacological management, containing the interventions these QIs include. Also, these QIs have been formulated in more detail, for example, regarding the specific content of self-management (eg, coping style with health problems). This may be due to the great focus on informing, advising and self-management in the Dutch KHOA guidelines for physiotherapy where these QIs are derived from. Second, QIs for centralised intake care in Canada^34^ is a healthcare setting that aims to prioritise access to care for patients with KHOA with a great focus on the structure of care. This is reflected by the fact that this study was the only one that included structure QIs. Third, QIs on pharmacological treatment were mainly described in studies about primary care setting. This is likely explained by the fact that primary care focuses on non-surgical treatment, containing pharmacological and non-pharmacological therapy, compared with secondary care. Altogether, the differences that this systematic review identified between QIs emphasise the heterogeneity of QIs for KHOA depending on the healthcare setting.

This systematic review did however identify differences which could not be fully explained by healthcare setting. These QIs concerned laboratory test in case of an NSAID prescription for ≥6 months to detect possible adverse events, a BMI threshold and frequency for advising patients to lose weight, and specific indications for radiographic assessment for KHOA. For example, two studies focusing on healthcare in the US described different indications, one describing that patients with incident hip OA should be offered an anteroposterior radiograph^39^ and another describing that patients with worsening complaints of KHOA accompanied by progressive decrease in activities should receive a radiograph within 3 months.^33^ However, this difference might be explained by the year of the study, which may indicate how up-to-date of the content of the QI is. The study describing that patients with worsening complaints should receive a radiograph^33^ was published more recently (ie, 2004) and is in line with the current evidence^51^ compared with the study that recommends a radiograph for patients with incident hip OA^39^ (ie, 2000). Another remarkable finding was that QIs on pharmacological treatment are consistent in the use of paracetamol as first-line pharmacological therapy and prescribing NSAIDs after a trial of maximum-dose paracetamol. However, recent guidelines do not recommend the use of paracetamol and the use of topical NSAIDs instead of paracetamol is strongly recommended.^8^ QIs about pharmacological treatment might be mostly influenced by guidelines and need to be up-to-date with the most recent guidelines. In addition, more agreement and uniformly formulated QIs within similar healthcare settings on these themes are needed to enhance uniform requirements for quality of care.

Of some frequently used treatments for OA, very little is described in QIs. For example, only one of 196 identified QIs focused on the prescription of opioids. Furthermore, QIs regarding injections, not prescribing chondroitin and glucosamine-chondroitin and indications for surgical treatment for hip OA are scarce. Also, there is currently an overuse of imaging to diagnose KHOA, while guidelines recommend to diagnose KHOA clinically.^5–7 52^ However, none of the studies focusing on primary care included QIs on imaging, while in these countries, the diagnosis and management of OA is mainly provided in primary care with general practitioners as the gatekeepers. Supplementing current QI sets, especially for primary care, with QIs on imaging may be helpful in reducing the overuse of imaging for the diagnosis of OA. In addition, although evidence shows the benefits of treatment tailored to patients’ preferences for satisfaction with treatment, uptake, and effectiveness of treatment,^53^ QIs relating to patients’ preferences are scarce. QIs mainly represented the perspective of healthcare professionals, while the perspectives of patients are just as important,^54^ as they are the service users of healthcare.^55^ Hence, future research on development of QIs on these themes is needed.

This systematic review was restricted to studies that developed QIs or adapted existing QIs. A previously published review^21^ on QIs for primary care for OA also included studies that evaluated the feasibility and reliability of existing QIs. We did not include these studies, while it may provide valuable information for the application of the QIs. We recommend for future research to evaluate implementation studies on the feasibility, validity and reliability of QI-sets in this review to add more guidance for the use of the QIs. Another limitation of this study may be that our literature search was not restricted on the date of publication, since our aim was to provide an extensive overview of the evidence. However, QIs from old studies may no longer apply to the current healthcare. Another limitation may be that we did not assess the quality of the included studies due to the absence of a quality assessment tool for studies developing QIs. To compensate the lack of such a tool, we presented the evidence synthesis and consensus method used in the included studies, which provided some information about the quality of the studies. Furthermore, we evaluated QIs from the literature using the Donabedian structure-process-outcome framework. However, other healthcare frameworks could have yielded other differences between healthcare settings and within the same healthcare settings. For example, the framework put forth by the Institute of Medicine, including the following six domains of quality of care: safe, effective, patient-centred, timely, efficient and equitable.^56^ Finally, our literature search did not include a search for websites for QIs in current use in quality or pay for performance programmes for specific hospitals or healthcare systems (eg, US National Quality Forum^57^ and UK National Institute for Health & Clinical Excellence^58^).

Previously published reviews^20 21 34 35 59 60^ focused on QIs specific healthcare settings (eg, primary care and centralised intake care systems), or perspectives (eg, patients’ perspectives). To our knowledge, this is the first systematic review that provides a comprehensive overview of QIs for KHOA outlining the differences and similarities between healthcare settings. This demonstrates the importance of selecting the contextually appropriate QIs to validly monitor the quality of care for KHOA.

## Conclusion

This review showed considerable differences between QIs depending on their healthcare settings. Furthermore, this review provides an overview of QIs outlined to their healthcare settings to support healthcare providers and policy makers in selecting the contextually appropriate QIs to validly monitor the quality of care for KHOA. However, we strongly recommend to review QIs against the most recent guidelines before implementing them into practice, especially QIs regarding pharmacological treatment. Furthermore, more adequate reporting of studies, rigorous methods of development of QIs and a greater variety of perspectives of stakeholders is needed. In addition, more uniformly formulated within the same healthcare settings and on several areas and up-to-date QIs are needed.
