# Supplementary material for: Quality indicators for knee and hip osteoarthritis care: a systematic review
Source: RMD Open. 2021 May 26;7(2):e001590. doi: 10.1136/rmdopen-2021-001590 (PMC8164978; doi:10.1136/rmdopen-2021-001590)
Supplement: Supplementary data [file rmdopen-2021-001590supp001.pdf]

Supplementary file 1: search strategy

Embase

('knee osteoarthritis'/de OR 'hip osteoarthritis'/de OR osteoarthritis/de OR (osteoarthrit* OR osteo-arthrit* OR osteoarthros* OR osteo-arthros* OR (degenerat* NEAR/3 (joint-disease* OR arthrit*)) OR ((knee OR hip OR knees OR hips) NEAR/6 (arthrosis* OR arthroses* OR arthrot* OR oa)) OR (oa AND rheuma*)):ab,ti) AND ('health care quality'/de OR 'total quality management'/de OR (((qualit* OR process OR performance* OR structure* OR outcome*) NEAR/3 (indicator* OR parameter*)) OR ((qualit*) NEAR/3 (care OR healthcare) NOT 'quality of life')):ab,ti)

Medline (Ovid)

(Osteoarthritis, Knee/ OR Osteoarthritis, Hip/ OR osteoarthritis/ OR (osteoarthrit* OR osteo-arthrit* OR osteoarthros* OR osteo-arthros* OR (degenerat* ADJ3 (joint-disease* OR arthrit*)) OR ((knee OR hip OR knees OR hips) ADJ6 (arthrosis* OR arthroses* OR arthrot* OR oa)) OR (oa AND rheuma*)).ab,ti.) AND (Quality of Health Care/ OR Total Quality Management/ OR Quality Indicators, Health Care/ OR (((qualit* OR process OR performance* OR structure* OR outcome*) ADJ3 (indicator* OR parameter*)) OR ((qualit*) ADJ3 (care OR healthcare) NOT quality of life)).ab,ti.)

CINAHL EBSCOhost

(MH Osteoarthritis, Knee OR MH Osteoarthritis, Hip OR MH osteoarthritis OR TI (osteoarthrit* OR osteo-arthrit* OR osteoarthros* OR osteo-arthros* OR (degenerat* N2 (joint-disease* OR arthrit*)) OR ((knee OR hip OR knees OR hips) N5 (arthrosis* OR arthroses* OR arthrot* OR oa)) OR (oa AND rheuma*)) OR AB (osteoarthrit* OR osteo-arthrit* OR osteoarthros* OR osteo-arthros* OR (degenerat* N2 (joint-disease* OR arthrit*)) OR ((knee OR hip OR knees OR hips) N5 (arthrosis* OR arthroses* OR arthrot* OR oa)) OR (oa AND rheuma*))) AND (MH Quality of Health Care OR MH Quality Improvement OR TI (((qualit* OR process OR performance* OR structure* OR outcome*) N2 (indicator* OR parameter*)) OR ((qualit*) N2 (care OR healthcare) NOT quality of life)) OR AB (((qualit* OR process OR performance* OR structure* OR outcome*) N2 (indicator* OR parameter*)) OR ((qualit*) N2 (care OR healthcare) NOT quality of life)))

Cochrane CENTRAL

((osteoarthrit* OR osteo-arthrit* OR osteoarthros* OR osteo-arthros* OR (degenerat* NEAR/3 (joint-disease* OR arthrit*)) OR ((knee OR hip OR knees OR hips) NEAR/6 (arthrosis* OR arthroses* OR arthrot* OR oa)) OR (oa AND rheuma*)):ab,ti) AND ((((qualit* OR process OR performance* OR structure* OR outcome*) NEAR/3 (indicator* OR parameter*)) OR ((qualit*) NEAR/3 (care OR healthcare) NOT 'quality of life')):ab,ti)

Web of science

TS=(((osteoarthrit* OR osteo-arthrit* OR osteoarthros* OR osteo-arthros* OR (degenerat* NEAR/2 (joint-disease* OR arthrit*)) OR ((knee OR hip OR knees OR hips) NEAR/5 (arthrosis* OR arthroses* OR arthrot* OR oa)) OR (oa AND rheuma*))) AND ((((qualit* OR process OR performance* OR structure* OR outcome*) NEAR/2 (indicator* OR parameter*)) OR ((qualit*) NEAR/2 (care OR healthcare) NOT "quality of life"))))

Google scholar

osteoarthritis|"osteo|degenerative arthritis|arthrosis|joint"|osteoarthrosis|"knee|hip|knees|hips arthrosis|arthroses|oa" "quality|process|performance|structure|outcome indicator|indicators|parameter|parameters"|"quality*care|healthcare"

Google

osteoarthritis|"osteo|degenerative arthritis|arthrosis|joint"|osteoarthrosis|"knee|hip|knees|hips arthrosis|arthroses|oa" "quality|process|performance|structure|outcome indicator|indicators|parameter|parameters"|"quality*care|healthcare" file-type:pdf

Open Grey

(osteoarthritis OR "degenerative arthritis") AND (quality) AND (indicator OR indicators OR parameter OR parameters)

Clinicaltrials.gov

(osteoarthritis OR "degenerative arthritis") AND (quality) AND (indicator OR indicators OR parameter OR parameters)

WHO ictrp

osteoarthritis AND quality AND indicator* OR osteoarthritis AND quality AND parameter* OR "degenerative arthritis" AND quality AND indicator* OR "degenerative arthritis" AND quality AND parameter* OR
