# Supplementary figures and images for: Quality indicators for knee and hip osteoarthritis care: a systematic review
Source: RMD Open. 2021 May 26;7(2):e001590. doi: 10.1136/rmdopen-2021-001590 (PMC8164978; doi:10.1136/rmdopen-2021-001590)

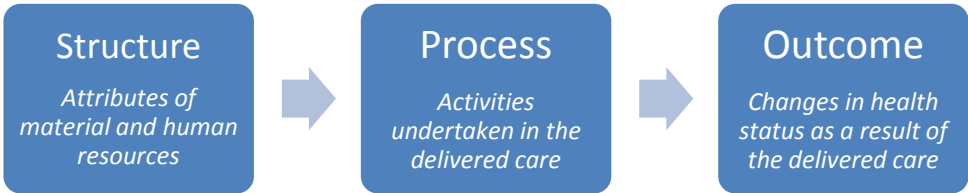

Figure 1. Donabedian’s structure-process-outcome quality of care model

Supplement: Supplementary data [file rmdopen-2021-001590supp002.pdf]
