# Supplementary material for: Quality indicators for knee and hip osteoarthritis care: a systematic review
Source: RMD Open. 2021 May 26;7(2):e001590. doi: 10.1136/rmdopen-2021-001590 (PMC8164978; doi:10.1136/rmdopen-2021-001590)
Supplement: Supplementary data [file rmdopen-2021-001590supp003.pdf]

Supplementary file 2: reasons of excluded studies on full text

Duplicate (n=8)

1. Edwards, J. J. (2017). Quality indicators for the care of osteoarthritis in general practice: identification, synthesis, and implementation, core.ac.uk.

2. Hay, E. M., et al. (2018). "Optimal primary care management of clinical osteoarthritis and joint pain in older people: a mixed-methods programme of systematic reviews, observational and …." … Grants for Applied ….
3. McLean, C. (2001). Quality indicators for the management of osteoarthritis in vulnerable elders ACOVE.
4. Petrosyan, Y., et al. (2018). "Quality indicators for ambulatory care for older adults with diabetes and comorbid conditions: A delphi study." PLoS ONE 13(12).
5. Račić, M., et al. (2016). "Quality of osteoarthritis care in family medicine – A cross-sectional study." Srp Arh Celok Lek 144(11-12): 633-638.
6. Silman, A. J. and G. A. Lkamh (2007). Quality of care for patients with osteoarthritis: a qualitative study.
7. Woolf, A. (2015). "SP0235 How to Develop Quality Indicators." Annals of the Rheumatic Diseases.
8. Olry de Labry Lima, A., et al. (2017). "Identification of health outcome indicators in Primary Care. A review of systematic reviews." Rev Calid Asist 32(5): 278-288.

Conference abstract (n=35)

1. Blackburn, S. J., et al. (2014). "Patients and researchers sometimes think alike: Patient involvement in the development of quality indicators for osteoarthritis care." Ann Rheum Dis 73.
2. Blackburn, S., et al. (2017). "Improving care for patients with osteoarthritis in five european countries: the jigsaw-e patient panel." Ann Rheum Dis 76: 1343-1344.
3. Borkhoff, C. M., et al. (2010). "Reaching those most in need: A scoping review of interventions to improve health care quality for disadvantaged populations with osteoarthritis." Osteoarthritis Cartilage 18: S163.
4. Buchbinder, R., et al. (2017). "Development of the osteoarthritis of the knee clinical care standard for clinicians and consumers." Intern Med J 47: 20.
5. Cavka, B., et al. (2018). "Implementing an ICHOM standard set to capture osteoarthritis outcomes in real world clinical settings." Osteoarthritis Cartilage 26: S267.

6. Dziedzic, K. (2016). "Getting evidence into practice: Pragmatic trials and implementation research." Osteoarthritis Cartilage 24: S3-S4.
7. Dziedzic, K., et al. (2016). "Joint implementation of guidelines for osteoarthritis in Western Europe: JIGSAW-E." Physiotherapy 102: e138-e139.
8. Edwards, J. J. (2015a). "An overview of quality indicators for the management of osteoarthritis in primary care." Ann Rheum Dis 74: 57-58.
9. Edwards, J. J., et al. (2015b). "Effect of a model consultation on quality of care of osteoarthritis: A primary care cluster randomised trial." Ann Rheum Dis 74: 108-109.
10. Gakhal, N. K., et al. (2016). "Audit and feedback of patient reported outcomes in knee osteoarthritis to improve management in primary care: A pilot project." Osteoarthritis Cartilage 24: S239-S240.
11. Grønhaug, G., et al. (2013). "Satisfaction and quality of hip and knee oa management in norwegian primary health care. Is the glass half full or half empty?" Ann Rheum Dis 72.
12. Grypdonck, L., et al. (2013). "Knee osteoarthritis: Widespread use of treatments that are not considered essential for high quality care." Ann Rheum Dis 72.
13. Hagen, K. B., et al. (2013). "Primary care for people with hip and knee osteoarthritis can be substantially improved. Results from a systematic review of studies comparing actual practice with desired practice." Ann Rheum Dis 71.
14. Kendzerska, T., et al. (2013). "A systematic review of the literature on best practice and quality of care for patients with osteoarthritis in the setting of diabetes or cardiovascular disease." Osteoarthritis Cartilage 21: S255-S256.
15. Kwoh, C. (2008). I-5 MOVING FROM GUIDELINES TO STANDARDS OF CARE, WB Saunders.
16. Li, L. (2015). "How can quality indicators be used to assess or monitor quality of care?" Ann Rheum Dis 74: 58.
17. Loza, E. (2015). "Quality indicators in rheumatology." Ann Rheum Dis 74: 57.
18. Marra, C., et al. (2011). "The pharmacist initiated intervention trial in osteoarthritis (PhIT-OA): Clinical outcomes." J Rheumatol 38(6): 1139
19. Nolan, G., et al. (2017). "Consumers' perspectives on current and future management of hip and/or knee osteoarthritis in Victoria: A consultation for the Victorian model of care for osteoarthritis of the hip and knee." Intern Med J 47: 10-11.
20. Østeras, N., et al. (2012). "Quality of osteoarthritis care: Test-retest reliability and feasibility of the osteoarthritis quality indicator questionnaire." Osteoarthritis Cartilage 20: S185-S186.

21. Østerås, N., et al. (2012). "Quality of osteoarthritis care: Test-retest reliability and feasibility of the osteoarthritis quality indicator questionnaire." Osteoarthritis Cartilage 20: S185-S186.
22. Østerås, N., et al. (2013). "Lower self-reported quality of care for persons with hand osteoarthritis compared to lower limb osteoarthritis." Ann Rheum Dis 72.
23. Østerås, N., et al. (2015). "Differences in self-reported quality of care for knee osteoarthritis across Denmark, Norway, Portugal and United Kingdom." Ann Rheum Dis 74: 326.
24. Østerås, N., et al. (2016). "Lower self-reported quality of care among persons with hand osteoarthritis compared to lower limb osteoarthritis-results from two cross-sectional observational studies." Osteoarthritis Cartilage 24: S38-S39.
25. Østerås, N., et al. (2018). "Implementing international osteoarthritis guidelines in an integrated care model e results from a cluster randomized controlled trial." Osteoarthritis Cartilage 26: S34-S35.
26. Peter, W. F. H., et al. (2011). "Development of quality indicators for physical therapy in hip and knee osteoarthritis." Arthritis Care Res 63(10).
27. Peter, W. F., et al. (2015). "Quality indicators for physiotherapy management in hip and knee osteoarthritis and rheumatoid arthritis; development and measurability." Ann Rheum Dis 74: 188.
28. Peter, W. F., et al. (2016). "Structure, process and outcome of primary care rheumatology networks for patients with rheumatic and musculoskeletal diseases in The Netherlands." Ann Rheum Dis 75: 1274-1275.
29. Petersson, I. F., et al. (2012). "Health Care Quality Indicators for Rheumatoid Arhtritis and Osteoarthritis:The Eumusc.Net Project." Ann. Rheum. Dis. 71: 621-621.
30. Quicke, J. G., et al. (2019). "Implementing and evaluating a pilot physiotherapist-led osteoarthritis clinic in general practice." Physiotherapy 105: e33-e34.
31. Santos-Moreno, P., et al. (2017). "Proposal for the constitution implementation and development of the program of centers of excellence in osteoarthritis in latin america." Ann Rheum Dis 76: 1342.
32. Schiphof, D., et al. (2017). "Joint implementation of guidelines for osteoarthritis in Western Europe: JIGSAW-E in progress in The Netherlands." Osteoarthritis and ….
33. Strömbeck, B., et al. (2013). "Health care quality indicators on the management of rheumatoid arthritis and osteoarthritis: A systematic review (in the framework of the eumusc.net project)." Ann Rheum Dis 71.
34. Wells, M., et al. (2016). "Quality measures in high priority rheumatologic diseases: A systematic literature review and analysis." Arthritis Rheum 68: 1758-1759.

35. Westby, M., et al. (2018). "Patients' resource preferences for a knowledge translation toolkit for hip and knee replacement rehabilitation." J Rheumatol 45(7): 990-991.
36. Woolf, A. (2015). "How to develop quality indicators." Ann Rheum Dis 74: 57.

Goal (n=64)

1. Alami, S., et al. (2011). "Patients' and practitioners' views of knee osteoarthritis and its management: A qualitative interview study." PLoS ONE 6(5).
2. Algeo, N., et al. (2017). "Usability of a digital self-management website for people with osteoarthritis: A UK patient and public involvement study." … Journal of Therapy ….
3. Allen, K. D., et al. (2016). "Osteoarthritis: Models for appropriate care across the disease continuum." Best Pract. Res. Clin. Rheumatol. 30(3): 503-535.
4. Askari, M., et al. (2011). "Assessing quality of care of elderly patients using the ACOVE quality indicator set: a systematic review." PLoS ONE 6(12): e28631.
5. Basedow, M. and A. Esterman (2015). "Assessing appropriateness of osteoarthritis care using quality indicators: A systematic review." J Eval Clin Pract 21(5): 782-789.
6. Brand, C. A., et al. (2013). "Chronic Disease Management. A Review of Current Performance Across Quality of Care Domains and Opportunities for Improving Osteoarthritis Care." Rheum Dis Clin North Am 39(1): 123-143.
7. Brand, C. A., et al. (2014). "Chronic disease management: improving care for people with osteoarthritis Review." Baillieres Best Pract Res Clin Rheumatol 28(1): 119-142.
8. Brosseau, L., et al. (2014). "A systematic critical appraisal for non-pharmacological management of osteoarthritis using the appraisal of guidelines research and evaluation II instrument Review." PLoS ONE 9(1): e82986.
9. Carr, E. C. J., et al. (2019). "Co-design of a patient experience survey for arthritis central intake: an example of meaningful patient engagement in healthcare design." BMC Health Serv. Res. 19.
10. Dateline, D. C. (2017). Patient-reported outcomes in surgery: listening to patients improves quality of care, bulletin.facs.org.
11. Dieppe, P. (2001). "From protocols to principles, from guidelines to toolboxes: Aids to good management of osteoarthritis." Rheumatology (UK) 40(8): 841-842.
12. Doerr, C. R., et al. (2013). "Implementation of a quality care management system for patients with arthritis of the hip and knee." Aust Health Rev 37(1): 88-92.
13. Dziedzic, K. S., et al. (2016). Implementation of musculoskeletal Models of Care in primary care settings: theory, practice, evaluation and outcomes for musculoskeletal health in high …, Elsevier.

14. Edwards, J. J., et al. A model consultation for osteoarthritis: the effect on the recorded quality of primary care.
15. Edwards, J. J., et al. (2014). "Quality of care for OA: The effect of a point-of-care consultation recording template." Rheumatology 54(5): 844-853.
16. Erwin, J., et al. (2017). "Better arthritis care: What training do community-based health professionals need to improve their care of people with arthritis? A Delphi study."
17. Ganz, D. A., et al. (2006). "Quality of osteoarthritis care for community-dwelling older adults." Arthritis Care Res 55(2): 241-247.
18. Glazier, R. H., et al. (2003). "Patient and provider factors related to comprehensive arthritis care in a community setting in Ontario, Canada." J Rheumatol 30(8): 1846-1850.
19. Gooch, K. L., et al. (2009). "The Alberta hip and knee replacement project: A model for health technology assessment based on comparative effectiveness of clinical pathways." Int J Technol Assess Health Care 25(2): 113-123.
20. Grønhaug, G., et al. (2015). "Perceived quality of health care services among people with osteoarthritis – Results from a nationwide survey." Patient Preference Adherence 9: 1255-1261.
21. Grønhaug, G., et al. (2014). "Quality of hip and knee osteoarthritis management in primary health care in a Norwegian county: a cross-sectional survey." BMC Health Serv Res 14: 598.
22. Hagen, K. B., et al. (2016). "Quality of Community-Based Osteoarthritis Care: A Systematic Review and Meta-Analysis." Arthritis Care Res 68(10): 1443-1452.
23. Higashi, T., et al. (2007). "Relationship between number of medical conditions and quality of care." New Engl J Med 356(24): 2496-2504.
24. Holm, I., et al. (2019). "A Pragmatic Approach to the Implementation of Osteoarthritis Guidelines Has Fewer Potential Barriers Than Recommended Implementation Frameworks." J Orthop Sports Phys Ther 49(1): 1-4.
25. Hunter, D. J. (2010). "Quality of osteoarthritis care for community-dwelling older adults." Clin Geriatr Med 26(3): 401-417.
26. Hunter, D. J., et al. (2011). "Quality of osteoarthritis management and the need for reform in the us." Arthritis Care Res 63(1): 31-38.
27. Hunter, D. J. and J. L. Bowden (2017). "Are you managing osteoarthritis appropriately?" Nature Reviews Rheumatology.
28. Ingelsrud, L. H., et al. (2020). "Patients report inferior quality of care for knee osteoarthritis prior to assessment for knee replacement surgery–a cross-sectional study of 517 patients in Denmark." Acta Orthop 91(1): 82-87.
29. Jackson, H., et al. (2017). "Patterns of routine primary care for osteoarthritis in the UK: A cross-sectional electronic health records study." BMJ Open 7(12).

30. Jansen, M. J., et al. (2005). "Treatment according to the Dutch guideline on osteoarthritis of the hip or knee: a prospective cohort study." NED TIJDSCHR FYSIOTHER 115(3): 68-73.
31. Jordan, K. P., et al. (2017). "Effect of a model consultation informed by guidelines on recorded quality of care of osteoarthritis (MOSAICS): a cluster randomised controlled trial in primary care." Osteoarthritis Cartilage 25(10): 1588-1597.
32. Kilic, Z., et al. (2019). "Benefits, Treatment Compliance, Awareness and Expectation Levels Related to Treatment of Patients Taking Physical Therapy Program." Turk Osteoporoz Derg. 25(3): 78-82.
33. Larmer, P. J., et al. (2019). "Quality indicators for hip and knee osteoarthritis management in New Zealand: A patient survey." NZ J PHYSIOTHER 47(3): 183-192.
34. Li, L. C., et al. (2011). "Quality of nonpharmacological care in the community for people with knee and hip osteoarthritis." J Rheumatol 38(10): 2230-2237.
35. Lin, I., et al. (2020). "What does best practice care for musculoskeletal pain look like? Eleven consistent recommendations from high-quality clinical practice guidelines: systematic review." Br J Sports Med 54(2): 79-86.
36. MacLean, C. H. (2001). "Evaluating the quality of care in rheumatic diseases." Current opinion in rheumatology.
37. Mann, C. and R. Gooberman-Hill (2011). "Health care provision for osteoarthritis: Concordance between what patients would like and what health professionals think they should have." Arthritis Care Res 63(7): 963-972.
38. McHugh, G. A., et al. (2012). "Quality of care for individuals with osteoarthritis: A longitudinal study." J Eval Clin Pract 18(3): 534-541.
39. McHugh, G. A., et al. (2007). "Quality of care for people with osteoarthritis: A qualitative study." J Clin Nurs 16(7 B): 168-176.
40. Miller, J. L., et al. (2016). "Support for Living a Meaningful Life with Osteoarthritis: A Patient-to-Patient Research Study." Patient 9(5): 457-464.
41. Nenova, G., et al. (2016). "Satisfaction of patients with arthrosis from multidisciplinary cooperation." J IMAB Ann Proc 22(4): 1338-1343.
42. Osajie, F. E. and K. Yakubu (2015). "A retrospective non-comparative analysis of the quality of care for osteoarthritis at the general out-patient department of Jos University Teaching Hospital, Nigeria." J. family med. prim. 4(2): 217-220.
43. Østerås, N., et al. (2019). "Implementing a structured model for osteoarthritis care in primary healthcare: A stepped-wedge cluster-randomised trial." PLoS Med 16(10).
44. Pincus, T. and T. Sokka (2009). "Quantitative clinical rheumatology: "Keep it simple, stupid": MDHAQ function, pain, global, and RAPID3 quantitative scores to improve and document the quality of rheumatologic care." J Rheumatol 36(6): 1099-1100.

45. Racic, M., et al. (2016). "Quality of osteoarthritis care in family medicine - A cross-sectional study." Srp. Ark. Celok. Lek. 144(11-12): 633-638.
46. Roberts, C., et al. (2002). "Improving the quality of care of musculoskeletal conditions in primary care." Rheumatology (UK) 41(5): 503-508.
47. Rodríguez, M. J., et al. (2018). "Evaluation of the quality of care of elderly patients with chronic and breakthrough pain treated with opioids: SAND study." Curr Med Res Opin 34(4): 701-709.
48. Rosemann, T., et al. (2006). "Problems and needs for improving primary care of osteoarthritis patients: the views of patients, general practitioners and practice nurses." BMC Musculoskelet. Disord. 7.
49. Saag, K. G., et al. (2011). Defining quality of care in rheumatology: the American College of Rheumatology white paper on quality measurement, Wiley Online Library.
50. Sampsel, S. L., et al. (2007). "Methods to develop arthritis and osteoporosis measures: A view from the National Committee for Quality Assurance (NCQA)." Clin Exp Rheumatol 25(6 SUPPL. 47): S22-S27.
51. Seibert, K., et al. (2019). "A systematic review on population-based indicators of the quality of care in formal and informal provider networks and their application in health economic evaluations." Z. Evid. Fortbild. Qual. Gesundh.wes. 144-145: 7-23.
52. Spitaels, D., et al. (2016). "Are physiotherapists adhering to quality indicators for the management of knee osteoarthritis? An observational study." Man Ther.
53. Spitaels, D., et al. (2017). "Barriers for guideline adherence in knee osteoarthritis care: A qualitative study from the patients' perspective." J Eval Clin Pract 23(1): 165-172.
54. Spitaels, D., et al. (2019). "Educational outreach visits to improve knee osteoarthritis management in primary care." BMC Med Educ 19(1): 66.
55. Spitaels, D., et al. (2019). "Quality of care for knee osteoarthritis in primary care: a patient's perspective." Arthritis Care Res (Hoboken).
56. Steel, N., et al. (2007). "Quality of clinical primary care and targeted incentive payments: An observational study." Br J Gen Pract 57(539): 449-454.
57. Steel, N., et al. (2014). "Self-reported quality of care for older adults from 2004 to 2011: A cohort study." Age Ageing 43(5): 716-720.
58. Teo, P. L., et al. (2019). "Identifying and Prioritizing Clinical Guideline Recommendations Most Relevant to Physical Therapy Practice for Hip and/or Knee Osteoarthritis." J. Orthop. Sports Phys. Ther. 49(7): 501-+.
59. Tornow, K., et al. (2019). "A Quality Improvement Project to Reduce Unnecessary Knee MRI for Chronic Degenerative Changes." J Am Coll Radiol 16(7): 940-944.

60. Umapathy, H., et al. (2015). "My joint pain: Web-based osteoarthritis management resource improves quality of care." Intern Med J 45: 18.
61. Vallejo-Torres, L. and S. Morris (2018). "Primary care supply and quality of care in England." Eur J Health Econ 19(4): 499-519.
62. Wang, X., et al. (2020). "My joint pain, a web-based resource, effects on education and quality of care at 24 months." BMC Musculoskelet Disord 21(1).
63. Westby, M. D., et al. (2016). "Emerging Role of Quality Indicators in Physical Therapist Practice and Health Service Delivery." Phys. Ther. 96(1): 90-100.
64. Westropp, J. C. (2002). "ACOVE: New tools address unmet need in quality assessment for older patients." Geriatrics 57(2): 44-51.

Goal and target group (n=6)

1. Black, N. and C. Jenkinson (2009). "How can patients' views of their care enhance quality improvement?" BMJ (Online) 339(7714): 202-205.
2. Curtis, J. R. and K. G. Saag (2004). "Evaluating and improving the quality of care in rheumatic disease." Expert Rev Pharmacoecon Outcomes Res 4(4): 429-439.
3. Dougados, M. (2007). "EULAR efforts to define quality of care." Clin. Exp. Rheumatol. 25(6): S14-S17.
4. Harrington, J. T. (2008). "Quality of care in rheumatic diseases: performance measures and improvement." CURR OPIN RHEUMATOL 20(2): 153-158.
5. Kazis, L. E., et al. (2006). "Dissemination of methods and results from the Veterans Health Study: Final comments and implications for future monitoring strategies within and outside the Veterans healthcare system." J AMBUL CARE MANAGE 29(4): 310-319.
6. Oostendorp, R. A. B., et al. (2019). "We are missing more. An international measurable model of clinical reasoning using quality indicators and routinely collected data." J. Man. Manip. Ther. 27(5): 253-257.

Protocol study (n=2)

1. Dziedzic, K. S., et al. (2014). "Implementing the NICE osteoarthritis guidelines: a mixed methods study and cluster randomised trial of a model osteoarthritis consultation in primary care--the Management of OsteoArthritis In Consultations (MOSAICS) study protocol." Implement Sci **9**: 95.
2. Østerås, N., et al. (2015). "Implementing international osteoarthritis treatment guidelines in primary health care: study protocol for the SAMBA stepped wedge cluster randomized controlled trial." Implement Sci **10**: 165.

Language (Spanish) (n=1)

1. Olry de Labry Lima, A., et al. (2017). "[Identification of health outcome indicators in Primary Care. A review of systematic reviews] Spanish." Rev. calid. asist. 32(5): 278-288.

QIs not presented (n=2)

1. Johansen, I., et al. (2019). "A quality indicator set for use in rehabilitation team care of people with rheumatic and musculoskeletal diseases; development and pilot testing." BMC Health Serv. Res. 19.
2. Yazdany, J. and C. H. MacLean (2008). "Quality of care in the rheumatic diseases: Current status and future directions." CURR OPIN RHEUMATOL 20(2): 159-166.

Target group (n=6)

1. Braun, J., et al. (2016). "Cornerstones of quality assurance in medicine in Germany. Important impulse for the situation in treatment of rheumatism." Z. Rheumatol. 75(2): 203-212.
2. Gnanadesigan, N. and C. Fung (2007). "Quality indicators for screening and prevention in vulnerable elders." Journal of the American Geriatrics Society.
3. Kerr, E. A., et al. (2000). Quality of care for general medical conditions: a review of the literature and quality indicators. dtic.mil.
4. MacLean, C. H., et al. (2000). "Quality of care for patients with rheumatoid arthritis." Jama.
5. Saag, K. G., et al. (2004). "Measuring quality in arthritis care: The arthritis foundation's quality indicator set for analgesics." Arthritis Care Res 51(3): 337-349.
6. Westby, M. D., et al. (2018). "Development of quality indicators for hip and knee arthroplasty rehabilitation." Osteoarthritis Cartilage 26(3): 370-382.

Reviews with no additional information about development, testing or implementation (feasibility) of QIs (n=6)

1. Edwards, J.J., et al. (2015). ‘’ Quality indicators for the primary care of osteoarthritis: a systematic review.’’ Ann Rheum Dis. 2015 Mar;74(3):490-8.
2. Falck, L., et al. (2019). "Toward Standardized Monitoring of Patients With Chronic Diseases in Primary Care Using Electronic Medical Records: Systematic Review." JMIR Med. Inf. 7(2): 136-148.
3. Hochberg, M. C. (2007). "Quality measures in osteoarthritis." Clin Exp Rheumatol 25(6 SUPPL. 47): S102-S106.
4. Petrosyan, Y., et al. (2017). "Quality indicators for care of osteoarthritis in primary care settings: a systematic literature review."

5. Petrosyan, Y. (2017). The quality of care among older adults with diabetes comorbid with other chronic conditions, tspace.library.utoronto.ca
6. Strömbeck, B., et al. (2013). "Health care quality indicators on the management of rheumatoid arthritis and osteoarthritis: A literature review." Rheumatology 52(2): 382-390.

Grey literature excluded on goal (n=1)

1. Quality of Care for Knee and Hip Osteoarthritis in Elderly Patients (QSAMISA). URL: https://clinicaltrials.gov/ct2/show/NCT04170218?cond=%28osteoarthritis+OR+%22degenerative+arthritis%22%29+AND+%28quality+%29+AND+%28indicator+OR+indicators+OR+parameter+OR+parameters%29&sfpd_s=10%2F12%2F2018&sfpd_e=04%2F07%2F2020&draw=2&rank=1
