# Supplementary material for: Quality indicators for knee and hip osteoarthritis care: a systematic review
Source: RMD Open. 2021 May 26;7(2):e001590. doi: 10.1136/rmdopen-2021-001590 (PMC8164978; doi:10.1136/rmdopen-2021-001590)
Supplement: Supplementary data [file rmdopen-2021-001590supp004.pdf]

Supplementary file 3: characteristics of included studies

Asch et al.(1)

General information	
Year	2004
Target population	Patient of a spectrum of outpatient and inpatient care (that is, screening, diagnosis, treatment, and follow-up) for acute and chronic conditions and preventive care processes representing the leading causes of morbidity, death, and health care use among older male patients (including patients with OA).
Setting/context/ health system	US Veterans Health Administration (VHA) health care systems
Study design	RAND approach/ modified Delphi method and cross-sectional comparison to evaluate quality of care.
Perspective of quality of care	Not specified.
Level of care of quality indicators	Process level of care
Proposed method of measurement of QIs	Medical records
Evidence synthesis	Review of national guidelines and the medical literature, but not systematically.
Consensus method	RAND approach/modified Delphi method.
Implementation of QIs	Asch et al.(1) implemented the QIs between 1997 and 2000 in 12 VHA health care systems and 12 communities in the US.
Testing of QIs	Quote: "Charts were reabstracted charts for 4% of the participants selected at random. According to the κ statistic, average reliability in the national sample was substantial to almost perfect at 3 levels: presence of a condition (κ = 0.83), indicator eligibility (κ = 0.76), and indicator scoring (κ = 0.80)"
Conflict of interest	No conflict of interest.
Adherence to the protocol	Unclear: no protocol published/reported.
Quality indicators*	
Providers caring for patients with symptoms of hip or knee osteoarthritis should recommend exercise programs at least once in 2 years.	
Patients with a new diagnosis of osteoarthritis who wish to take medication for joint symptoms should be offered a trial of acetaminophen.	
Providers caring for patients with symptoms of osteoarthritis should document all of the following at least once in 2 years: the location of symptoms and/or the presence or absence of limitations in daily activities.	

**Note: The presented QIs are developed for a broader spectrum of patient than only OA patient. For the current review, only the indicators regarding OA were extracted.*

Barber et al.(2)

General information	
Year	2015
Target population	Rheumatoid arthritis (RA) patients and OA patients (patients with moderate to severe OA who required either surgical (total hip or knee arthroplasty) or nonsurgical management (requiring specialist consultation)).
Setting/context/ health system	Centralize intake care system in Canada.
Study design	Stakeholder meetings, literature review and Delphi rounds: a modification of the RAND-UCLA Appropriateness Method.
Perspective of quality of care	Healthcare professionals, organizational and patients.
Level of care of quality indicators	Structure, process, and outcome level of care
Proposed method of measurement of QIs	Not reported.
Evidence synthesis	Integrative review including an update of a systematic review of the literature conducted by the European Musculoskeletal Conditions Surveillance and Information Network in two literature databases (MEDLINE and Embase) to identify all existing performance measures for OA and RA.
Consensus method	Stakeholder meetings and Delphi rounds: a modification of the RAND-UCLA Appropriateness Method.
Implementation of QIs	Assessment of feasibility (i.e. how likely it is that the information required to report on the indicator will be available in the health system) done by an expert panel during the Delphi rounds.
Testing of QIs	Not done.
Conflict of interest	No conflict of interest.
Adherence to the protocol	Unclear: no protocol published/reported.

Quality indicators*
Time from OA referral receipt to referral completion for initially incomplete referrals.
Time from receipt of complete OA referral to musculoskeletal appointment.
Distribution of OA referrals in each urgency category (as scored using the Western Canada Waiting List referral tool).
Percentage of OA referrals triaged as highest urgency based on high Western Canada Waiting List priority criteria scores seen within Wait Time Alliance benchmarks.
Percentage of referrals rejected or redirected when received at centralized intake.
Percentage of OA referrals received with complete information.
Percentage of OA referrals scored using Western Canada Waiting List priority referral criteria.
Number of referrals received through centralized intake.
Agreement of centralized intake suspected diagnosis of severe OA cases (e.g., patients who are candidates for hip or knee joint replacements) versus confirmed diagnosis of severe OA.
Percentage of patients who receive information regarding resources and tools available for management while waiting for first musculoskeletal specialty contact.
Operating room time for arthroplasty surgeons in Alberta.
Percentage of specialist providers participating in centralized intake.
Musculoskeletal specialty care provider experience with centralized intake.
Administrative staff and allied health professional experience with centralized intake.

Referring clinician's experience with centralized intake.
Percentage of musculoskeletal appointments completed as scheduled.
Ratio of patient flow to estimated clinic capacity of OA teams participating in centralized intake.
Patient experience with centralized intake.

**Note: QIs for OA specific and OA + rheumatoid arthritis were extracted (thus; QIs for only RA were excluded)*

Blackburn et al.(3)

General information	
Year	2017
Target population	Patients with OA.
Setting/context/ health system	Primary care in the UK.
Study design	Discussion meetings with a literature review.
Perspective of quality of care	Patients.
Level of care of quality indicators	Process and outcome level of care
Proposed method of measurement of QIs	Patient-reported questionnaire. Intended for use in the Management of OSteoArthritis In ConsultationS (MOSAICS) study, which developed and evaluated a new model of supported self-management of OA to implement the NICE guidelines.
Evidence synthesis	The authors used information from an earlier published systematic review(4), but did not conduct a literature review.
Consensus method	Four discussion groups with the research team to develop QIs.
Implementation of QIs	Not done.
Testing of QIs	Not done.
Conflict of interest	No conflict of interest.
Adherence to the protocol	Unclear: no protocol published/reported.
Quality indicators*	
You have been offered information or advice on exercise or activity to help with your joint problem.	
You have received advice about body weight and joint pain.	
You have received advice and support on how you might help yourself to manage or deal with your joint problem.	
You have been offered advice about medications (to relieve joint pain).	
You have been offered a referral to an exercise or activity program for your joint problem.	
You have been offered a referral for physiotherapy for your joint problem.	
You have received a referral for weight loss services.	
You have been given a follow-up review of your joint problem.	
You are satisfied with the overall quality of the consultation with his/her GP for OA.	

**Note: Two QIs were developed regarding postoperative treatment of osteoarthritis. With respect to the exclusion criteria of our review, these QIs were excluded, thus not presented in this overview.*

Broadbent et al.(5)

General information	
Year	2008
Target population	Patients with OA.
Setting/context/ health system	Primary care: UK general practice.
Study design	Development of indicators and retrospective observational study.
Perspective of quality of care	Not reported/specified.
Level of care of quality indicators	Process level of care
Proposed method of measurement of QIs	Medical records.
Evidence synthesis	The authors included QIs that were based on the following sources: NICE; RAND health indicators adapted by an independent expert panel including British GPs for the UK (Steel et al.(6)), and Quality Indicators for General Practice developed at the National Primary Care Research and Development Centre.
Consensus method	Not reported.
Implementation of QIs	Broadbent et al.(5) implemented the QIs in eighteen general practices in the UK.
Testing of QIs	Not done.
Conflict of interest	Unclear: Nicholas Steel was funded by a Primary Care Researcher Development Award from the UK National Coordinating Centre for Research Capacity Development (RDA03/21). Unclear whether this organisation has its certain interests or benefits with this study.
Adherence to the protocol	Unclear: no protocol published/reported.

Quality indicators
The percentage of patients with symptomatic osteoarthritis, whose notes contain a record that they have been offered education regarding the natural history, treatment, and self-management of the disease at least once.
The percentage of patients in whom oral pharmacological therapy was initiated to treat osteoarthritis, whose notes contain a record that they were offered paracetamol first (unless contraindicated).
The percentage of patients with osteoarthritis treated with an NSAID, whose notes contain a record that ibuprofen (or a cox-2 inhibitor) has been considered for first-line treatment (unless contraindicated or intolerant)
The percentage of patients in whom oral pharmacological therapy was changed from paracetamol to a different oral agent, whose notes contain a record that they were offered a trial of maximum-dose paracetamol.
The percentage of patients with osteoarthritis treated with an NSAID, whose notes contain a record that they have been advised of the gastrointestinal and renal risks associated with this drug.
The percentage of patients with osteoarthritis regularly treated with an NSAID, whose notes contain a record that they have been asked about gastrointestinal symptoms within the previous 12 months.
The percentage of patients with severe symptomatic osteoarthritis of the knee or hip that has failed to respond to non-pharmacological and pharmacological therapy, whose notes contain a record that they were offered referral to an orthopaedic surgeon to be evaluated for total joint replacement within 6 months unless surgery is contraindicated.
The percentage of patients treated for symptomatic osteoarthritis, whose notes contain a record that they

have been assessed for functional status in the last year.
The percentage of patients treated for symptomatic osteoarthritis, whose notes contain a record that they have been assessed for degree of pain in the last year.

Doubova et al.(7)

General information	
Year	2015
Target population	Patients with knee and hip OA older than 19.
Setting/context/ health system	Primary care: family medicine in Mexico.
Study design	Modified version of RAND-UCLA method (development of indicators) and a cross-sectional analysis (of quality-of-care provided for patients with osteoarthritis).
Perspective of quality of care	Healthcare professional.
Level of care of quality indicators	Process level of care
Proposed method of measurement of QIs	Electronic health records.
Evidence synthesis	Literature review of scientific evidence in the following databases: Medline, Ovid, Cochrane Library, National Guideline Clearinghouse, CMA Infobase: Clinical Practice Guidelines, TRIP database, Institute for Clinical System Improvement, ACP Guideline website, American Academy of Family Physicians, NHS Evidence - National Library of Guidelines and IMSS-Clinical Guidelines. The literature search and review was performed by one researcher. No systematic review.
Consensus method	Modified version of the RAND/UCLA Appropriateness Method
Implementation of QIs	Doubova et al.(7) implemented the QIs in four family medicine clinics in Mexico City.
Testing of QIs	Not done.
Conflict of interest	No conflict of interest.
Adherence to the protocol	Unclear: no protocol published/reported.
Quality indicators	
(Patients with knee/hip OA who have documented recommendations for general aerobic and/or muscle strengthening exercise at least once per year, unless contraindicated (e.g. significant heart failure)/ Total number of patients with KHOA without contraindications for general aerobic exercise) * 100	
(Overweight (BMI ≥27 kg/m2) patients with KHOA who have documented nutritional counselling provided by the Nutrition and Dietary Service and/or who were encouraged by their family physician at least one time per year to lose weight/ Total number of overweight patients with KHOA) * 100	
(Patients with newly diagnosed of KHOA who received prescription of acetaminophen as initial oral analgesic, unless* contraindicated/ Total number of patients with recent diagnosis of KHOA) * 100	
(Patients aged 65 years or older with KHOA and one of the following comorbidities (history of peptic ulcer disease or gastrointestinal bleeding, chronic kidney disease, cardiac insufficiency and/or those receiving anticoagulant or glucocorticoids) who receive NSAID prescription/ Total number of patients aged 65 years or older with KHOA and one of the previously mentioned comorbidities)* 100	
(Patients with KHOA and high risk for gastrointestinal complications who received NSAID prescription	

concomitant with either misoprostol or a proton-pump inhibitor/ Total number of patients with KHOA and high risk of gastrointestinal complications who received NSAIDs) * 100
(Patients with KHOA and with NSAID prescription for 6 months or longer who were referred for the following laboratory tests (blood count, serum creatinine and liver enzymes) at least once in the previous 12 months / Total number of patients with KHOA and with NSAID prescription for 6 months or longer) * 100

** Note: originally, this quality indicator formulated instead of 'unless' the word 'otherwise'. In order to present the indicator in the similar way as the other indicators of this study and make the interpretation easier, we contacted the author of this study and changed the word 'otherwise' into 'unless' with the authors permission.*

Grypdonck et al.(8)

General information	
Year	2014
Target population	Patients with knee OA.
Setting/context/ health system	The entire spectrum of disciplines involved in knee OA care.
Study design	RAND-modified Delphi method.
Perspective of quality of care	Healthcare professional.
Level of care of quality indicators	Process level of care.
Evidence synthesis	Literature was searched in PubMed, Embase, and the World Wide Web (English and Dutch) for existing guidelines and sets of quality indicators. Unclear whether this was done systematically.
Consensus method	RAND-modified Delphi method.
Proposed method of measurement of QIs	Not identified.
Implementation of QIs	Not done.
Testing of QIs	Not done.
Conflict of interest	Unclear.
Adherence to the protocol	Unclear: no protocol published/reported.
Quality indicators	
If a patient has knee OA, then exercise therapy should be prescribed, including at least muscle strengthening, aerobic exercises and functional exercises, and combined with range of motion exercises in case of range of motion restrictions.	
If a patient with knee OA is following exercise therapy, then the content and intensity of the exercise program should be tailored to the patient's individual goals in terms of limitations of activity and restrictions of participation.	
If a patient with knee OA is following exercise therapy, then the treatment sessions should be spread over longer periods with lower frequencies in the later stages of the exercise program to facilitate the transition from exercise therapy to independent exercising and maintaining sufficient level of physical activity.	
If a patient with knee OA is following exercise therapy, then he/she should be referred to regular community exercise and sports activities after a period of supervised exercise.	
If a patient with knee OA is following exercise therapy, then regular evaluations by the physiotherapist are	

necessary. To make the switchover from a supervised to an autonomous program, an evaluation session should be performed every 3 months in the first year, every 6 months in the second year, and once per year afterward.
If a patient with knee OA is following exercise therapy, then the exercise therapy should be combined with education/self-management interventions to improve patients' mental and physical performance and to alleviate pain.
If a patient with knee OA is overweight, then he/she should be encouraged to lose weight and maintain his/her weight at a lower level.
If a patient has knee OA, he/she should be given information access and education about the objectives of treatment and the importance of changes in lifestyle, exercise, pacing of activities, weight reduction, and other measures to unload the damaged joints.
If a patient has knee OA, then acetaminophen up to 3 g/day should be used as the initial oral analgesic.
If a patient has knee OA and there is no adequate response on acetaminophen, or there is severe pain and/or inflammation, then oral NSAID should be used.
If a patient has knee OA, then chondroitin and glucosamine-chondroitin combination products should not be used.
If NSAID are used in a patient with knee OA, then they should be used intermittently (max 3 weeks sustained use) and at the lowest effective dose.
If a patient with knee OA and a history of bleeding gastric ulcers has a need for NSAID, then either a COX-2 selective agent or a non-selective NSAID with coprescription of a proton pump inhibitor/misoprostol should be used instead of a non-selective NSAID.
If a patient with knee OA has heart failure grade 2–4, ischemic heart disease, or renal insufficiency with a GFR < 40 ml/min, then NSAID should not be used. In case of other cardiovascular risk factors (e.g., hypertension, …), NSAID should be used with caution.
If a patient has knee OA, then strong opioids (oxymorphone, oxycodone, fentanyl, morphine sulfate) should not be used.
If a patient has symptomatic knee OA, then he/she has to be referred to a physical therapist for instruction of the patient in appropriate exercises, for motivation of the patient to implement exercise and adhere to exercise, and to evaluate performance.
If a patient with knee OA is not obtaining adequate pain relief and functional improvement, then he/she should be considered for joint replacement.
If a patient has unicompartmental knee OA, then a unicompartmental knee replacement should be considered.
If a patient has knee OA, then arthroscopic interventions are not recommended. Coexisting meniscal lesions should not be treated. Only in case of locking of the knee from a large meniscal fragment or a loose body or an extension loss from an anterior anvil osteophyte is arthroscopic treatment indicated.
If a patient is clinically diagnosed with knee OA and suffering from pain resistant to conservative treatment with acetaminophen and/or NSAID, then a radiography (weight-bearing, semiflexed PA, plus lateral and skyline view) of the symptomatic knee should be taken for the morphological assessment and grading of knee OA (especially to detect unicompartmental OA, for which treatment modalities may differ). CT and MRI scan should not be used.
If a patient with knee OA has a recurrent clinically evident effusion, then he/she should be further assessed (with aspiration and analysis of synovial fluid) in order to differentiate from inflammation caused by other arthritis.
If a patient has knee OA, then a brace should not be prescribed (except in unicompartmental knee OA with axial deviation).

Hardcastle et al.(9)

General information	
Year	2015
Target population	People with OA aged 50 years or older living in private households in England.
Setting/context/ health system	UK health system.
Study design	Adaptation of QIs by a modified RAND/UCLA appropriateness method and quality measurement using face-to-face interviews.
Perspective of quality of care	Healthcare professional.
Level of care of quality indicators	Process level of care
Evidence synthesis	Authors used QIs of Steel et al.(6); only difference is that they took a subset that would be feasible for surveys and adapted the age from 65 into 50.
Consensus method	Modified RAND/UCLA appropriateness method.
Proposed method of measurement of QIs	Patient interview surveys.
Implementation of QIs	Assessment for feasibility of survey use by an expert panel of clinicians.
Testing of QIs	Not done.
Conflict of interest	No conflict of interest
Adherence to the protocol	Unclear: no protocol published/reported.
Quality indicators*	
IF an ambulatory person aged ≥ 50 years has had a diagnosis of symptomatic osteoarthritis of the knee for longer than 3 months and has no contraindications to exercise and is physically and mentally able to exercise, THEN a directed or supervised strengthening or aerobic exercise programme should have been prescribed at least once.	
IF an ambulatory person aged ≥ 50 years has a diagnosis of symptomatic osteoarthritis, THEN education regarding the natural history, treatment and self-management of the disease should be offered at least once.	
IF oral pharmacological therapy is initiated to treat osteoarthritis among people aged ≥ 50 years, THEN paracetamol should be the first drug used, unless there is a contraindication to use.	
IF a person aged ≥ 50 years with severe symptomatic osteoarthritis of the knee or hip has failed to respond to non-pharmacological and pharmacological therapy, THEN the patient should be offered referral to an orthopaedic surgeon to be evaluated for total joint replacement within 6 months unless surgery is contraindicated.	

**Note: The presented QIs were developed for a broader spectrum of patient than only OA patient. For the current review, only the indicators regarding OA were extracted.*

Jansen et al. (10)

General information	
Year	2010
Target population	Patients with knee and/or hip OA.
Setting/context/ health system	Physiotherapy care in the Netherlands.
Study design	Prospective cohort study.

Perspective of quality of care	Researchers; list of QIs was made by the authors of this article.
Level of care of quality indicators	Process and outcome level of care.
Evidence synthesis	No literature review performed in the study. QIs were derived from the Dutch physiotherapy guideline on hip and knee OA.
Consensus method	The process and outcome indicators were formulated by one authors, and independently assessed by two other authors. The process indicators were derived from the key recommendations in the guidelines. Not reported were the outcome indicators come from.
Proposed method of measurement of QIs	Physiotherapist self-reported recording forms. Not reported whether this was online or on paper.
Implementation of QIs	27 physical therapists recorded patient and treatment characteristics of at least five consecutive patients with knee and hip osteoarthritis. Problems with filling in/the use of the form were discussed afterwards.
Testing of QIs	Not done.
Conflict of interest	Unclear: this study was funded by a grant from the Royal Dutch Society of Physical Therapy (KNGF), however, no statement has been made regarding their involvement with the conduct of the study and interpretation and reporting of the results.
Adherence to the protocol	Unclear: no protocol published/reported.
Quality indicators	
Problem areas recorded (i.e. inflammation, pain, impairments of function, activity limitations, participation restrictions, and passive coping behaviour) (benchmark >90%)	
Patient profile recording according to the Dutch physiotherapy guidelines (benchmark >90%)	
Measurements of the VAS for severity of pain and Algofunctional Index measurements at baseline, at 6 weeks and at the end of the treatment episode (benchmark >90%)	
Information and advice (benchmark >90%)	
Exercise therapy for body functions (benchmark >90%)	
Exercise therapy for activities (benchmark >90%)	
No massage therapy (benchmark <10%)	
No use of physical modalities other than TENS (e.g. pulsed shortwave) (benchmark <10%)	
Aftercare (e.g. home exercise programme, follow up consultation, advice to participate in community based or sport programmes) (benchmark >90%)	
VAS for severity of pain decrease of more than 25%	
Algofunctional index decrease of more than 25%	
The extent to which the treatment goals were achieved*	
Number of sessions lower than 12	
Duration of treatment episode less than 6 weeks	
Treatment frequency *	
Patients satisfaction with treatment*	
Global perceived effect either for pain or for restrictions in daily activities (5 point Likert scale)*	

* No specific threshold reported in the article; QIs are developed based on the Dutch physiotherapy guideline and further information on thresholds is documented in the recommendations of this guideline.

MacLean et al.(11) (ACOVE-1)

General information	
Year	2001
Target population	Vulnerable elders with OA.
Setting/context/ health system	US health system.
Study design	Systematic review and RAND/UCLA Appropriateness Method.
Perspective of quality of carePerspective of quality of care	Not reported/specified.
Level of care of quality indicators	Process level of care.
Evidence synthesis	Systematic review.
Consensus method	Modified RAND/UCLA Appropriateness Method.
Proposed method of measurement of QIs	Medical records, administrative data, and/or patient or proxy interview.
Implementation of QIs	Not done.
Testing of QIs	Not done.
Conflict of interest	No conflict of interest.
Adherence to the protocol	Unclear: no protocol published/reported.
Quality indicators	
IF an ambulatory vulnerable elder is newly diagnosed with osteoarthritis of the knee, has no contraindication to exercise, and is physically and mentally able to exercise, THEN a directed or supervised strengthening or aerobic exercise program should be prescribed within 3 months of diagnosis BECAUSE such programs improve functional status and reduce pain.	
IF an ambulatory vulnerable elder has had a diagnosis of symptomatic osteoarthritis of the knee for longer than 12 months, has no contraindication to exercise, and is physically and mentally able to exercise, THEN there should be evidence that a directed or supervised strengthening or aerobic exercise program was prescribed at least once since the time of diagnosis BECAUSE such programs improve functional status and reduce pain.	
IF an ambulatory vulnerable elder is diagnosed with symptomatic osteoarthritis THEN education regarding the natural history, treatment, and self-management of the disease should be offered at least once within 6 months of diagnosis BECAUSE such education produces improvements in physical functioning and pain.	
IF a patient COX has had a diagnosis of symptomatic osteoarthritis for 12 months or longer THEN there should be evidence that the patient was offered education regarding the natural history, treatment, and selfmanagement of the disease at least once since the time of diagnosis BECAUSE such education produces improvements in physical functioning and pain.	
IF oral pharmacologic therapy is initiated to treat osteoarthritis in a vulnerable elder, THEN acetaminophen should be the first drug used, unless there is a documented contraindication to use, BECAUSE this agent is as effective in treating osteoarthritis as other oral agents, and it is less toxic.	
IF oral pharmacologic therapy for osteoarthritis in a vulnerable elder is changed from acetaminophen to a different oral agent, THEN there should be evidence that the patient has had a trial of maximum-dose acetaminophen (suitable for age and comorbid conditions) BECAUSE acetaminophen, in adequate doses, is as effective in treating osteoarthritis as other oral agents, and it is less toxic.	
IF a patient is treated with a COX nonselective nonsteroidal anti-inflammatory drug (NSAID), THEN there should be evidence that the patient was advised of the risk for gastrointestinal bleeding associated with these drugs	

BECAUSE this risk is substantial.
IF a vulnerable elder is older than 75 years of age, is treated with warfarin, or has a history of peptic ulcer disease or gastrointestinal bleeding, AND is being treated with a COX nonselective NSAID, THEN he or she should be offered concomitant treatment with either misoprostol or a proton-pump inhibitor BECAUSE this will substantially reduce the risk for NSAID-induced gastrointestinal bleeding.
IF a vulnerable elder with severe symptomatic osteoarthritis of the knee or hip has failed to respond to nonpharmacologic and pharmacologic therapy and has no contraindication to surgery, THEN the patient should be referred to an orthopaedic surgeon to be evaluated for total joint replacement within 6 months unless a contraindication to surgery is documented BECAUSE hip and knee replacements markedly improve function and quality of life by reducing pain and/or improving range of motion.
IF a vulnerable elder is diagnosed with symptomatic osteoarthritis, THEN his or her functional status and the degree of pain should be assessed annually BECAUSE this information is necessary to direct therapeutic decisions.
IF a vulnerable elder has monoarticular joint pain associated with redness, warmth, or swelling AND the patient also has an oral temperature greater than 38.0 °C and does not have a previously established diagnosis of pseudogout or gout, THEN a diagnostic aspiration of the painfully swollen red joint should be performed that day BECAUSE this sign–symptom complex is common with joint infection, and it requires treatment that is different than that for osteoarthritis.

MacLean et al.(12) (ACOVE-2)

General information	
Year	2004
Target population	Individuals with OA.
Setting/context/ health system	US health system.
Study design	Comprehensive literature review and modified RAND/UCLA Appropriateness Method.
Perspective of quality of care	Not reported/specified.
Level of care of quality indicators	Process level of care.
Evidence synthesis	Systematic review.
Consensus method	Modified RAND/UCLA appropriateness method.
Proposed method of measurement	Medical records, administrative data, and/or patient or proxy interview.
Implementation of QIs	Not done.
Testing of QIs	Not done.
Conflict of interest	No conflict of interest.
Adherence to the protocol	Unclear: no protocol published/reported.
Quality indicators	
IF an ambulatory patient has had a diagnosis of symptomatic osteoarthritis of the knee or hip for >3 months AND has no contraindication to exercise and is physically and mentally able to exercise, THEN a directed or supervised muscle strengthening or aerobic exercise program should have been prescribed at least once and reviewed at least once per year.	
IF an individual is overweight (as defined by body mass index of ≥27 kg/m2), THEN the individual should be advised to lose weight annually.	

IF a patient has symptomatic osteoarthritis of the knee or hip and is overweight (as defined by body mass index of ≥27 kg/m2), THEN the patient should be advised to lose weight at least annually AND the benefit of weight loss on the symptoms of osteoarthritis should be explained to the patient.
IF a patient has had a diagnosis of symptomatic osteoarthritis of the knee or hip for >3 months, THEN education about the natural history, treatment, and self-management of osteoarthritis should have been given or recommended at least once.
IF a nonnarcotic pharmacologic therapy is initiated to treat osteoarthritis pain of mild or moderate severity, THEN acetaminophen should be the first drug used, unless there is a documented contraindication to use.
IF oral pharmacologic therapy for osteoarthritis is changed from acetaminophen to a different oral agent, THEN there should be evidence that the patient has had a trial of maximum-dose acetaminophen (suitable for age/comorbidities).
IF a patient with severe symptomatic osteoarthritis of the knee or hip has failed to respond to nonpharmacologic and pharmacologic therapy, THEN the patient should be offered referral to an orthopedic surgeon.
IF a patient has hip or knee osteoarthritis AND has worsening complaints accompanied by a progressive decrease in activities AND no previous radiograph during the preceding 3 months, THEN a knee or hip radiograph should be performed within 3 months.
IF a patient has symptomatic osteoarthritis of the knee or hip and has been overweight (as defined by body mass index of ≥27 kg/m2) for >3 years, THEN the patient should receive referral to a weight loss program.
IF a patient is begun on a drug treatment for joint pain, arthritis, or arthralgia, THEN evidence that the affected joint was examined should be documented.
IF a patient is diagnosed with symptomatic osteoarthritis of the knee or hip, THEN his or her pain should be assessed annually and when new to a practice.
IF a patient is diagnosed with symptomatic osteoarthritis of the knee or hip, THEN his or her functional status should be assessed annually and when new to a practice.
IF a patient has had symptomatic osteoarthritis of the hip or knee and reports difficulty walking to accomplish activities of daily living for >3 months, THEN the patient's walking ability should be assessed for need of ambulatory assistive devices.
IF a patient has a diagnosis of osteoarthritis and reports difficulties with nonambulatory activities of daily living, THEN the patient's functional ability with problem tasks should be assessed for need of nonambulatory assistive devices to aid with problem tasks.

MacLean et al.(13) (ACOVE-3)

General information	
Year	2007
Target population	Vulnerable elders: These are community-dwelling individuals aged 65 and older who are at greater risk of death or functional decline over a 2-year period.
Setting/context/ health system	US health system.
Study design	Systematic review and modified RAND/UCLA Appropriateness Method.
Perspective of quality of care	Healthcare professionals.
Level of care of quality indicators	Process level of care
Evidence synthesis	Systematic review.

Consensus method	Modified RAND/UCLA appropriateness method.
Proposed method of measurement of QIs	Medical records, administrative data, and/or patient or proxy interview.
Implementation of QIs	Not done.
Testing of QIs	Not done.
Conflict of interest	No conflict of interest
Adherence to the protocol	Unclear: no protocol published/reported.
Quality indicators*	
IF an ambulatory vulnerable elder (VE) has symptomatic OA of the knee or hip for longer than 3 months and is able to exercise, THEN a directed or supervised muscle strengthening or aerobic exercise program should be recommended and activity reviewed annually.	
IF a VE is started on pharmacological therapy to treat OA, THEN acetaminophen should be tried first.	
IF a VE is prescribed chronic high-dose acetaminophen (≥3 g/d) or a VE with liver disease is prescribed chronic acetaminophen, THEN he or she should be advised of the risk of liver toxicity, BECAUSE these risks are greater with high doses of acetaminophen and when underlying liver disease is present.	
IF a VE is prescribed an NSAID (non- selective or selective), THEN GI bleeding risks should be discussed and documented.	
IF a VE is prescribed daily aspirin (including low-dose,<325mg/d), THEN GI bleeding risks should be discussed and documented, BECAUSE selective NSAIDs, non-selective NSAIDs, and aspirin increase the risk of bleeding.	
IF a VE with a risk factor for GI bleeding (aged ≥75, peptic ulcer disease, history of GI bleeding, warfarin use, chronic glucocorticoid use) is treated with a nonselective NSAID, THEN he or she should be treated concomitantly with misoprostol or a proton pump inhibitor (PPI).	
IF a VE with two or more risk factors for GI bleeding (aged ≥75, peptic ulcer disease, history of GI bleeding, warfarin use, chronic glucocorticoid use) is treated with daily aspirin, THEN he or she should be treated concomitantly with either misoprostol or a PPI, BECAUSE this will reduce the risk of GI bleeding.	
IF a VE has severe symptomatic OA of the knee or hip despite nonsurgical therapy, THEN a referral to an orthopedic surgeon should be made.	
IF a VE has symptomatic OA of the knee or hip, THEN pain should be assessed when new to a primary care or musculoskeletal disease practice and annually.	
IF a VE has symptomatic OA of the knee or hip, functional status should be assessed when new to a primary care or musculoskeletal disease practice and annually, BECAUSE this information should direct therapeutic decisions.	
IF a VE has symptomatic OA of the hip or knee and has difficulty walking that makes activities of daily living difficult for longer than 3 months, THEN the need for ambulatory assistive devices should be assessed.	
IF a VE has symptomatic OA and has difficulty with nonambulatory activities of daily living, THEN the need for activity of daily living assistive devices should be assessed.	
IF a VE is obese (body mass index (BMI) ≥30kg/m2), THEN he or she should be advised annually to lose weight, BECAUSE weight loss reduces the risk of developing symptomatic knee and hip OA.	

**Note: The presented QIs were developed for a broader spectrum of patient than only OA patient. For the current review, only the indicators regarding OA were extracted.*

Marshall et al.(14)

General information	
Year	2003
Target population	Patients with osteoarthritis.
Setting/context/ health system	General practices in the UK healthcare system.
Study design	Literature review, RAND/UCLA Appropriateness Method, and field-testing of indicators using electronic and paper records in general practices.
Perspective of quality of care	Healthcare professionals.
Level of care of quality indicators	Process level of care
Evidence synthesis	Literature review; not systematically.
Consensus method	RAND/UCLA appropriateness method.
Proposed method of measurement of QIs	Electronic and paper records from the general practice.
Implementation of QIs	Field-testing on 1600 randomly selected patient records in 16 general practices belonging to two demographically contrasting English Primary Care Trusts.
Testing of QIs	Unclear, reliability of QIs was tested for diseases other than OA in this study.
Conflict of interest	No conflict of interest
Adherence to the protocol	Unclear: no protocol published/reported.
**Quality indicators**	
Patients with a new diagnosis of osteoarthritis who wish to take medication for joint symptoms should be offered a trial of paracetamol if not already tried.	
If NSAIDs are considered, Ibuprofen should be considered for first line treatment unless contraindicated or intolerant.	
Patients with osteoarthritis prescribed oral NSAIDs who are at high risk of gastrointestinal side effects (past history of dyspepsia or known peptic ulcer) should be considered for a co-prescription of PPIs, H2 antagonists or Misoprolol, unless contraindicated or intolerant	
Patients with severe symptomatic osteoarthritis of the knee or hip who have failed to respond to conservative therapy should be offered referral to an orthopaedic surgeon for consideration of joint replacement.	

**Note: The presented QIs are developed for a broader spectrum of patient than only OA patient. For the current review, only the indicators regarding OA were extracted.*

Moore et al.(15)

General information	
Year	2000
Target population	Patients with osteoarthritis.
Setting/context/ health system	US healthcare system.
Study design	Literature review and RAND/UCLA Appropriateness Method.
Perspective of quality of care	Healthcare professionals.
Level of care of quality indicators	Process level of care.

Evidence synthesis	Literature review: reviewed relevant textbooks and review articles in MEDLINE with a basic strategy.<br>No systematic review.
Consensus method	RAND/UCLA appropriateness method.
Proposed method of measurement of QIs	Medical records.
Implementation of QIs	Assessment for feasibility of the QIs was done by the expert panel.
Testing of QIs	Not done.
Conflict of interest	Unclear.
Adherence to the protocol	Unclear: no protocol published/reported.
Quality indicators	
Providers caring for patients with symptoms of OA should document all of the following at least once in 2 years:<br>a. the location of symptoms;<br>b. the presence or absence of limitations in daily activities;<br>c. the presence or absence of a history or symptoms of systemic or inflammatory disease;<br>d. the use and effectiveness of treatment modalities.	
Providers caring for patients with incident symptoms of OA should document at least one of the following:<br>• the presence or absence of a history of any systemic or inflammatory disease that may mimic OA;<br>• the presence or absence of any current symptoms of systemic or inflammatory disease that may mimic OA;<br>• the presence or absence of a history of joint trauma or surgery.	
Providers caring for patients with symptoms of OA should document the following for any one affected joint at least once in 2 years:<br>a. the presence or absence of effusion;<br>b. the presence or absence of bony enlargement;<br>c. the presence or absence of tenderness;<br>d. the presence or absence of limitations in range of motion.	
Patients with incident symptoms of hip OA should be offered an anteroposterior film of the affected hip.	
Patients with a new diagnosis of OA who wish to take medication for joint symptoms should be offered a trial of acetaminophen.	
Providers caring for patients with symptoms of hip or knee OA should recommend both of the following at least once in 2 years:<br>a. exercise programs for persons with hip or knee OA;<br>b. weight loss among persons with knee OA and a BMI >25.	
Patients receiving care for symptoms of OA should be seen in follow-up at least every 6 months.	

Østerås et al.(16)

General information	
Year	2018
Target population	Patients with OA.
Setting/context/ health system	Norwegian healthcare system.
Study design	Longitudinal, observational cohort study
Perspective of quality of care	Researchers: QIs were developed and assessed by researchers.

Level of care of quality indicators	Process level of care.
Evidence synthesis	Literature review, no systematic review.<br>Quote: "Studies reporting QIs for OA care published between 2000 and 2010 were identified via structured searches of 4 electronic databases (Medline, Embase, CINAHL, and AMED) using the search terms quality of health care, standards of care, quality indicators (Health Care), performance indicator, guidelines (Standards), osteoarthritis, degenerative arthritis, and arthritis care. The searches resulted in 565 potentially relevant articles. The first author (NØ) screened titles and abstracts, and 26 articles were read in full text." (Osteras 2013)
Consensus method	Revised the QI questionnaire of Østerås et al. (2013) which was developed through critical judgement by researchers working within rheumatology and having experience with questionnaire design. During 2010-2014 the first author (NØ) systematically collected and registered feedback from national and international colleagues that used the questionnaire in different settings. The experiences were critically reviewed and discussed. Thereafter, the expert group and patient research partners collaborated on developing a revised version.
Proposed method of measurement of QIs	Patient self-reported questionnaire.
Implementation of QIs	Feasibility of QIs V1 was assessed using patient questionnaires from 359 persons in a Norwegian OA cohort. The revised version, the OA-QI v2, was then pilot-tested by 11 of the members in the Patient Research Partner Panel at Diakonhjemmet Hospital, who had no comments on the wording revisions that were done.
Testing of QIs	Questionnaire test-retest κ=0.38–0.85, % exact agreement from 69–92%. The ICC for all 16 items was 0.89.
Conflict of interest	Unclear.
Adherence to the protocol	Unclear: no protocol published/reported.
Quality indicators	
Have you been given information about osteoarthritis from a health professional?	
Have you been given information about different treatment alternatives?	
Have you been given information about how you can self-manage the disease?	
Have you been given information about the importance of physical activity and exercise?	
Have you been advised to lose weight, if you are overweight?	
If you use anti-inflammatory medications, have you been given information about the effects and possible side-effects of this medication? (e.g. ibuprofen (Nurofen, Brufen), diclofenac (Voltarol), naproxen (Naprosyn),celecoxib (Celebrex))	
Have you been referred or offered a referral to a health professional who can advise you about physical activity and exercise?	
Have you been referred or offered a referral to someone who can help you to lose weight, if you are overweight?	
If you are severely troubled by your osteoarthritis, and exercise and medication do not help, have you been referred or offered a referral for an assessment for operation? (e.g. joint replacement)	

If you have problems with daily activities, have these problems been assessed by a health professional?
If you have problems with walking, has your need for a walking aid been assessed? (e.g. stick, crutch or walker)
If you have problems related to other daily activities, has your need for appliances and aids been assessed? (e.g. splints, assistive technology for cooking or personal hygiene, a special chair)
If you have joint pain, has it been assessed by a health professional?
If you have joint pain, was paracetamol the first medication that was recommended?
If you have prolonged severe joint pain, which is not relieved sufficiently by paracetamol, have you been offered stronger pain killing medications? (e.g. co-codamol, codeine, tramadol, co-proxamol, co-dydramol, dihydrocodeine)
If you have experienced an acute deterioration of your symptoms, have you been given or offered a steroid injection?

Peter et al.(17)

General information	
Year	2013
Target population	Patients with knee and/or hip OA.
Setting/context/ health system	Physiotherapy care in the Netherlands.
Study design	Expert panel methods and cross-sectional implementation of the QI-questionnaire.
Perspective of quality of care	Healthcare providers
Level of care of quality indicators	Process level of care.
Evidence synthesis	No literature review performed in the study. QIs were derived from the Dutch physiotherapy guideline on hip and knee OA.
Consensus method	Rating of recommendations of guideline by an expert panel of physical therapists in primary and secondary care with respect to its potential contribution to quality of care, acceptability and measurability for daily practice. The resulting recommendations were transformed to quality indicators.
Proposed method of measurement of QIs	Physiotherapist self-reported online questionnaire.
Implementation of QIs	The QI-questionnaire was pilot-tested with respect to clarity and completeness by 15 PTs working in primary care and three experts in the development of tests. Consecutively, a pilot test was also done among expert (n= 51) and general (n = 134) PTs and 58 PTs who were considered to be neither expert nor general PTs in the Netherlands.
Testing of QIs	All participating PTs were sent a hyperlink to the online version of the questionnaire by email. Participants were invited to complete the questionnaire at two different time points, within seven days, to determine the test–retest reliability. ICC was 0.89, meaning that the QI-questionnaire was found to be reliable.
Conflict of interest	Unclear.
Adherence to the protocol	Unclear: no protocol published/reported.

Quality indicators
Inventory of health-related problems according to the International Classification of Functioning, Disability and Health (ICF)
Assessing the presence of personal and environmental problems in so far as these relate to the limitations in activities and restrictions in participation
Assessing the presence of hip and knee OA-specific 'red flags'
Treating patients with strengthening of muscles
Treating patients with improving of aerobic capacity
Treating patients with walking exercises
Treating patients with functional exercises
Providing information concerning knowledge and understanding of OA of the hip and/or knee}
Providing information concerning the consequences for the patient's functional performance in terms of movements, activities and participation}
Providing information concerning the relationship between burden and tolerance level
Providing information concerning the way a patient copes with health problems
Providing information concerning what constitutes an active and healthy lifestyle (in terms of exercise and nutrition/overweight
Providing information concerning behavioural change (regarding physical activity)
Providing information concerning joint protection and the use of aids
Evaluating treatment with the recommended measurement instruments
Evaluating treatment with the combination of a questionnaire and a performance test
Evaluating treatment with a patient-specific complaint list
Evaluating treatment with the Timed Up and Go test (TUG)

** Note: One QI was developed regarding postoperative treatment of knee and hip osteoarthritis. With respect to the exclusion criteria of our review, this QI was excluded, thus not presented in this overview.*

Saliba et al.(18)

General information	
Year	2004
Target population	Institutionalized vulnerable elders (including patient with OA).
Setting/context/ health system	Secondary care: nursing homes in the US.
Study design	Modified Delphi process.
Perspective of quality of care	Not reported/specified.
Level of care of quality indicators	Process level of care.
Evidence synthesis	No literature review. Adapted the ACOVE-1 set for the use in nursing homes in the US.
Consensus method	Modified Delphi process.
Proposed method of measurement of QIs	Not specified.
Implementation of QIs	Not cone.

Testing of QIs	Not done.
Conflict of interest	Unclear.
Adherence to the protocol	Unclear: no protocol published/reported.
Quality indicators*	
IF an ambulatory NH resident is newly diagnosed with symptomatic osteoarthritis of the knee and has no contraindication to exercise and is physically and mentally able to exercise THEN a directed or supervised strengthening or aerobic exercise program should be prescribed within 1 month of diagnosis.	
IF an ambulatory NH resident has a diagnosis of symptomatic knee osteoarthritis for >3 months, has no contraindication to exercise, and is physically and mentally able to exercise THEN there should be evidence that a directed or supervised strengthening or aerobic exercise program was prescribed at least once since the time of diagnosis.	
IF a non-OTC drug is newly prescribed to treat new joint pain THEN evidence that the affected joint was examined should be documented within 4 weeks.	
IF oral pharmacologic therapy is initiated to treat osteoarthritis THEN acetaminophen should be the first drug used.	
IF oral pharmacologic therapy for symptomatic osteoarthritis is changed from acetaminophen to a different oral agent THEN there should be evidence that the NH resident has had a trial of maximum dose acetaminophen (suitable for age and comorbid conditions).	
IF a NH resident has a new joint pain that is reported to the primary care provider THEN the joint and periarticular structures should be examined within 1 month or there should be documentation that the problem has resolved.	
IF a NH resident has monoarticular joint pain associated with redness, warmth, and/or swelling and the patient also has an oral temperature >38.0°C, and does not have a previously established diagnosis of pseudogout or gout THEN a diagnostic aspiration of the painfully swollen red joint should be performed that day.	

** Note: The presented QIs are developed for a broader spectrum of patient than only OA patient. For the current review, only the indicators regarding OA were extracted.*

Smith et al.(19)

General information	
Year	2007
Target population	Home-based primary care patients (including patients with OA).
Setting/context/ health system	Primary care to homebound seniors in the US.
Study design	A modified Delphi process.
Perspective of quality of care	Not reported/specified.
Level of care of quality indicators	Process level of care.
Evidence synthesis	No literature review. Adapted the ACOVE-1 set for the use in home-based primary care in the US.
Consensus method	Modified Delphi process.
Proposed method of measurement of QIs	Not specified.
Implementation of QIs	Not done.
Testing of QIs	Not done.

Conflict of interest	No conflict of interest.
Adherence to the protocol	Unclear: no protocol published/reported.
Quality indicators*	
IF an ambulatory homebound patient is newly diagnosed with osteoarthritis of the knee, has no contraindication to exercise, and is physically and mentally able to exercise, THEN a directed or supervised strengthening or aerobic exercise program should be prescribed within 3 months of diagnosis.	
IF an ambulatory homebound patient has had a diagnosis of symptomatic osteoarthritis of the knee for longer than 12 months and is physically and mentally able to exercise, THEN there should be evidence that a physical therapy evaluation for focused strengthening exercises was prescribed at least once since the time of diagnosis.	
IF oral pharmacologic therapy is initiated to treat osteoarthritis in a homebound patient, THEN acetaminophen should be the first drug used, unless there is a documented contraindication to use	
IF oral pharmacologic therapy for osteoarthritis in a homebound patient is changed from acetaminophen to a different oral agent, THEN there should be evidence that the patient has had a trial of maximum-dose acetaminophen (suitable for age and comorbid conditions).	
IF a patient is treated with a COX-nonselective NSAID, THEN there should be evidence that the patient was advised of the risk for gastrointestinal bleeding, as well as cardiovascular risk associated with these drugs.	
IF a homebound patient is diagnosed with symptomatic osteoarthritis, THEN his or her functional status and the degree of pain should be assessed at each visit.	
IF a homebound patient has monoarticular joint pain associated with redness, warmth, or swelling AND the patient also has an oral temperature greater than 38.0 °C and does not have a previously established diagnosis of pseudogout or gout, THEN diagnostic aspiration of the painfully swollen, red joint should be performed that day.	

**Note: The presented QIs are developed for a broader spectrum of patient than only OA patient. For the current review, only the indicators regarding OA were extracted.*

Steel et al.(6)

General information	
Year	2004
Target population	Older adults (people aged 65 years and older in England) (including patients with OA).
Setting/context/ health system	Primary and secondary care in the UK.
Study design	Modified RAND/UCLA appropriateness method.
Perspective of quality of care	Not reported/specified.
Level of care of quality indicators	Process level of care.
Evidence synthesis	No literature review. Adapted the ACOVE-1 set for use in UK healthcare system (translation from US to UK).
Consensus method	Modified RAND/UCLA appropriateness method.
Proposed method of measurement of QIs	Medical records.
Implementation of QIs	Not done.

Testing of QIs	Not done.
Conflict of interest	No conflict of interest.
Adherence to the protocol	Unclear: no protocol published/reported.
Quality indicators*	
IF an ambulatory person aged 65 or older has had a diagnosis of symptomatic osteoarthritis of the knee for longer than 3 months and has no contraindications to exercise and is physically and mentally able to exercise, THEN a directed or supervised strengthening or aerobic exercise programme should have been prescribed at least once.	
IF an ambulatory person aged 65 or older has a diagnosis of symptomatic osteoarthritis, THEN education regarding the natural history, treatment and self-management of the disease should be offered at least once.	
IF oral pharmacological therapy is initiated to treat osteoarthritis among people aged 65 or older, THEN paracetamol should be the first drug used, unless there is a contraindication to use.	
IF oral pharmacological therapy for osteoarthritis is changed from paracetamol to a different oral agent among people aged 65 or older, THEN the patient should have had a trial of maximum dose paracetamol (suitable for age/co-morbidities).	
IF a person aged 65 or older with severe symptomatic osteoarthritis of the knee or hip has failed to respond to non- pharmacological and pharmacological therapy, THEN the patient should be offered referral to an orthopaedic surgeon to be evaluated for total joint replacement within 6 months unless surgery is contraindicated.	
IF a person aged 65 or older is treated for symptomatic osteoarthritis, THEN functional status and degree of pain should be assessed at least annually.	

** Note: The presented QIs are developed for a broader spectrum of patient than only OA patient. For the current review, only the indicators regarding OA were extracted.*

VandenBerghe et al.(20)

General information	
Year	2004
Target population	Patients with osteoarthritis of 60 years or above in Belgium.
Setting/context/ health system	General practice in Belgium (primary care)
Study design	Cross-sectional study.
Perspective of quality of care	Not reported/specified.
Level of care of quality indicators	Process level of care.
Evidence synthesis	Unclear: method of derivation of QIs not described. Only described that the QIs originate from guidelines, but not specified which guidelines.
Consensus method	Unclear: method of derivation of QIs not described.
Proposed method of measurement of QIs	Either on paper registration sheets (paper group) or through an extraction of data from the electronic patient record (EPR group) by GPs
Testing of QIs	The quality indicators were implemented in Belgium in 2001 and 2003 in the general practices and data were compared between a pooled database (consultations and home visits) and a restricted database (after removal of home visits).
Conflict of interest	Not done.
Adherence to the protocol	Unclear: no statement regarding conflict of interest has been made in the

	article.
Testing of QIs	Unclear: no protocol published/reported.
Quality indicators	
Patients with a drug prescription for osteoarthritis in the past month (numerator)/ all patients with osteoarthritis (denominator)	
Patients who were prescribed paracetamol (numerator)/ all patients with a drug prescription for osteoarthritis in the past month (denominator)	
Patients which were prescribed an NSAID (numerator)/ all patients with a drug prescription for osteoarthritis in the past month (denominator)	
Patients who were prescribed a coxib (numerator)/ all patients who received an NSAID for osteoarthritis in the past month (denominator)	
Patients who received a repeated prescription/ all patients who received an NSAID for osteoarthritis in the past month (denominator)	

Wierenga et al.(21)

General information	
Year	2011
Target population	Elderly hospitalized patients in the Netherlands (including patients with OA).
Setting/context/ health system	Dutch in-hospital pharmaceutical care; secondary care.
Study design	Expert panel review methods.
Perspective of quality of care	Not reported/specified.
Level of care of quality indicators	Process level of care.
Evidence synthesis	No literature review. Adapted the ACOVE-1 set for use in Dutch in-hospital pharmaceutical care.
Consensus method	Expert panel review methods.
Proposed method of measurement of QIs	Medical records and a hospital information system.
Testing of QIs	Assessment for feasibility was done by the expert panel with ten preselected elderly patients who had experienced a long hospital stay, multiple co-morbidities and geriatric problems.
Conflict of interest	Quote: “The inter-rater agreement (reliability) was determined based on three pharmacists’ (YB, JK, MT) assessment of the quality of care of ten randomly selected patients (different to those used for the improvement of the QI phrasing).”<br>κ = 0.88 (95%CI=0.75, 1.00)<br>ICC= 0.80 (95%CI=0.63, 0.90)
Adherence to the protocol	No conflict of interest.
Testing of QIs	Unclear: no protocol published/reported.
Quality indicators*	
IF oral pharmacological therapy is initiated to treat osteoarthritis in an elder, THEN paracetamol (acetaminophen) should be the first drug used, UNLESS there is a documented contra-indication.	

IF oral pharmacological therapy for osteoarthritis in an elder is changed from paracetamol (acetaminophen) to a different oral agent, THEN there should be evidence that the patient has had a trial of maximum dose of paracetamol (suitable for age and co-morbid conditions.

**Note: The presented QIs are developed for a broader spectrum of patient than only OA patient. For the current review, only the indicators regarding OA were extracted.*

REFERENCES

1. Asch SM, McGlynn EA, Hogan MM. Comparison of quality of care for patients in the Veterans Health Administration and patients in a national sample: Am Coll Physicians; 2004.
2. Barber CE, Patel JN, Woodhouse L, Smith C, Weiss S, Homik J, et al. Development of key performance indicators to evaluate centralized intake for patients with osteoarthritis and rheumatoid arthritis. Arthritis Res Ther. 2015;17:322.
3. Blackburn S, Higginbottom A, Taylor R, Bird J, Osteras N, Hagen KB, et al. Patient-reported quality indicators for osteoarthritis: a patient and public generated self-report measure for primary care. Res Involv Engagem. 2016;2:5.
4. Edwards JJ, Khanna M, Jordan KP, Jordan JL, Bedson J, Dziedzic KS. Quality indicators for the primary care of osteoarthritis: a systematic review. Ann Rheum Dis. 2015;74(3):490-8.
5. Broadbent J, Maisey S, Holland R, Steel N. Recorded quality of primary care for osteoarthritis: An observational study. Br J Gen Pract. 2008;58(557):839-43.
6. Steel N, Melzer D, Shekelle PG. Developing quality indicators for older adults: transfer from the USA to the UK is feasible. Quality and Safety …. 2004.
7. Doubova SV, Perez-Cuevas R. Quality of care for hip and knee osteoarthritis at family medicine clinics: Lessons from Mexico. Int J Qual Health Care. 2015;27(2):125-31.
8. Grypdonck L, Aertgeerts B, Luyten F, Wollersheim H, Bellemans J, Peers K, et al. Development of quality indicators for an integrated approach of knee osteoarthritis. J Rheumatol. 2014;41(6):1155-62.
9. Hardcastle AC, Mounce LTA, Richards SH, Bachmann MO, Clark A, Henley WE, et al. The dynamics of quality: a national panel study of evidence-based standards. Health Services and Delivery Research. 2015;No. 3.11.
10. Jansen MJ, Hendriks EJ, Oostendorp RA, Dekker J, De Bie RA. Quality indicators indicate good adherence to the clinical practice guideline on "Osteoarthritis of the hip and knee" and few prognostic factors influence outcome indicators: a prospective cohort study. Eur J Phys Rehabil Med. 2010;46(3):337-45.
11. MacLean CH. Quality indicators for the management of osteoarthritis in vulnerable elders. Ann Intern Med. 2001;135(8 I):711-21.
12. MacLean CH, Saag KG, Solomon DH, Morton SC, Sampsel S, Klippel JH. Measuring Quality in Arthritis Care: Methods for Developing the Arthritis Foundation's Quality Indicator Set. Arthritis Care Res. 2004;51(2):193-202.
13. MacLean CH, Pencharz JN, Saag KG. Quality indicators for the care of osteoarthritis in vulnerable elders. J Am Geriatr Soc. 2007;55 Suppl 2:S383-91.
14. Marshall MN, Shekelle PG, McGlynn EA, Campbell S, Brook RH, Roland MO. Can health care quality indicators be transferred between countries? Qual Saf Health Care. 2003;12(1):8-12.
15. Moore A. Quality of Care for General Medical Conditions: Chapter 17. OSTEOARTHRITIS TREATMENT: RAND Corporation; 2000.

16. Osterås N, Garratt A, Grotle M, Natvig B, Kjeken I, Kvien TK, et al. Patient-reported quality of care for osteoarthritis: Development and testing of the osteoarthritis quality indicator questionnaire. Arthritis Care Res. 2013;65(7):1043-51.
17. Peter WF, van der Wees PJ, Hendriks EJ, de Bie RA, Verhoef J, de Jong Z, et al. Quality indicators for physiotherapy care in hip and knee osteoarthritis: development and clinimetric properties. Musculoskeletal Care. 2013;11(4):193-202.
18. Saliba D, Solomon D, Rubenstein L, Young R, Schnelle J, Roth C, et al. Quality indicators for the management of medical conditions in nursing home residents. J Am Med Dir Assoc. 2004;5(5):297-309.
19. Smith KL, Soriano TA, Boal J. Brief communication: National quality-of-care standards in home-based primary care. Ann Intern Med. 2007;146(3):188-92.
20. Vandenberghe H, Van Casteren V, Jonckheer P, Lafontaine MF, De Clercq E. Quality of care assessment using GPs' electronic patient records: do we need data from home visits? Stud Health Technol Inform. 2004;110:35-41.
21. Wierenga PC, Klopotowska JE, Smorenburg SM, Van Kan HJ, Bijleveld YA, Dijkgraaf MG, et al. Quality indicators for in-hospital pharmaceutical care of dutch elderly patients: Development and validation of an ACOVE-based quality indicator set. Drugs Aging. 2011;28(4):295-304.
