# Supplementary material for: Quality indicators for knee and hip osteoarthritis care: a systematic review
Source: RMD Open. 2021 May 26;7(2):e001590. doi: 10.1136/rmdopen-2021-001590 (PMC8164978; doi:10.1136/rmdopen-2021-001590)
Supplement: Supplementary data [file rmdopen-2021-001590supp005.pdf]

Supplementary Table 4. Quality indicators per category and theme

Theme	Quality indicator	Author	OA in the knee, hip, or not related to specific sites	Healthcare setting
**Structure**				
Musculoskeletal appointments	Percentage of musculoskeletal appointments completed as scheduled	Barber et al. (2016)	OA not related to specific sites	Centralized intake care system in Canada
Healthcare providers involved	Percentage of specialist providers participating in centralized intake.	Barber et al. (2016)	OA not related to specific sites	Centralized intake care system in Canada
Estimation of clinic capacity	Ratio of patient flow to estimated clinic capacity of OA teams participating in centralized intake.	Barber et al. (2016)	OA not related to specific sites	Centralized intake care system in Canada
**Process: history taking and examination**				
Regular assessment of functional status	The percentage of patients treated for symptomatic osteoarthritis, whose notes contain a record that they have been assessed for functional status in the last year.	Broadbent et al. (2008)	OA not related to specific sites	Primary care in the UK general practice
Regular assessment of pain	The percentage of patients treated for symptomatic osteoarthritis, whose notes contain a record that they have been assessed for degree of pain in the last year.	Broadbent et al. (2008)	OA not related to specific sites	Primary care in the UK general practice
Regular assessment of pain	IF a VE has symptomatic OA of the knee or hip, THEN pain should be assessed when new to a primary care or musculoskeletal disease practice and annually.	MacLean et al. (2007)	Hip/knee OA	US healthcare system
Regular assessment of pain	If you have joint pain, has it been assessed by a health professional?	Østerås et al. (2018)	OA not related to specific sites	Norwegian healthcare system
Regular assessment of pain and functional status	IF a vulnerable elder is diagnosed with symptomatic osteoarthritis, THEN his or her functional status and the degree of pain should be assessed annually BECAUSE this information is necessary to direct therapeutic decisions.	MacLean et al. (2001)	OA not related to specific sites	US healthcare system

Regular assessment of pain and functional status	IF a patient is diagnosed with symptomatic osteoarthritis of the knee or hip, THEN his or her pain should be assessed annually and when new to a practice.	MacLean et al. (2004)	Hip/knee OA	US healthcare system
Regular assessment of pain and functional status	IF a homebound patient is diagnosed with symptomatic osteoarthritis, THEN his or her functional status and the degree of pain should be assessed at each visit.	Smith et al. (2007)	OA not related to specific sites	Home-based primary care setting in the US
Regular assessment of pain and functional status	IF a person aged 65 or older is treated for symptomatic osteoarthritis, THEN functional status and degree of pain should be assessed at least annually.	Steel et al. (2004)	OA not related to specific sites	Primary and secondary care setting in the UK
Regular assessment of pain and functional status	IF a NH resident has a new joint pain that is reported to the primary care provider THEN the joint and periarticular structures should be examined within 1 month or there should be documentation that the problem has resolved.	Saliba et al. (2004)	OA not related to specific sites	Nursing homes in the US
Assessment for aid	If you have problems with walking, has your need for a walking aid been assessed? (e.g. stick, crutch or walker)	Østerås et al. (2018)	OA not related to specific sites	Norwegian healthcare system
Assessment for appliances and aids	If you have problems related to other daily activities, has your need for appliances and aids been assessed? (e.g. splints, assistive technology for cooking or personal hygiene, a special chair)	Østerås et al. (2018)	OA not related to specific sites	Norwegian healthcare system
Assessment for assistive devices	IF a VE has symptomatic OA of the hip or knee and has difficulty walking that makes activities of daily living (ADLs) difficult for longer than 3 months, THEN the need for ambulatory assistive devices should be assessed, BECAUSE these devices will improve walking and help maintain function and independence.	MacLean et al. (2007)	Knee OA	US healthcare system
Assessment for assistive devices	IF a VE has symptomatic OA and has difficulty with nonambulatory ADLs, THEN the need for ADL assistive devices should be assessed, BECAUSE such devices will help to maintain function and independence.	MacLean et al. (2007)	Knee OA	US healthcare system
Assessment for assistive devices	IF a patient has had symptomatic osteoarthritis of the hip or knee and reports difficulty walking to accomplish activities of daily living for 3 months, THEN the patient's walking	MacLean et al. (2004)	Knee/hip OA	US healthcare system

	ability should be assessed for need of ambulatory assistive devices.			
Assessment for assistive devices	IF a patient has a diagnosis of osteoarthritis and reports difficulties with nonambulatory activities of daily living, THEN the patient's functional ability with problem tasks should be assessed for need of nonambulatory assistive devices to aid with problem tasks.	MacLean et al. (2004)	OA not related to specific sites	US healthcare system
Radiographs	Patients with incident symptoms of hip OA should be offered an anteroposterior film of the affected hip.	Moore et al. (2000)	OA not related to specific sites	US healthcare system
Radiographs	IF a patient has hip or knee osteoarthritis AND has worsening complaints accompanied by a progressive decrease in activities AND no previous radiograph during the preceding 3 months, THEN a knee or hip radiograph should be performed within 3 months.	MacLean et al. (2004)	Hip/knee OA	US healthcare system
Radiograph	If a patient is clinically diagnosed with knee OA and suffering from pain resistant to conservative treatment with acetaminophen and/or NSAID, then a radiography (weight-bearing, semiflexed PA, plus lateral and skyline view) of the symptomatic knee should be taken for the morphological assessment and grading of knee OA (especially to detect unicompartmental OA, for which treatment modalities may differ). CT and MRI scan should not be used.	Grypdonck et al. (2014)	Knee OA	Entire spectrum of disciplines
Diagnostic aspiration	IF a vulnerable elder has monoarticular joint pain associated with redness, warmth, or swelling AND the patient also has an oral temperature greater than 38.0 °C and does not have a previously established diagnosis of pseudogout or gout, THEN a diagnostic aspiration of the painfully swollen red joint should be performed that day BECAUSE this sign–symptom complex is common with joint infection, and it requires treatment that is different than that for osteoarthritis.	MacLean et al. (2001)	OA not related to specific sites	US healthcare system

Diagnostic aspiration	IF a NH resident has monoarticular joint pain associated with redness, warmth, and/or swelling and the patient also has an oral temperature >38.0°C, and does not have a previously established diagnosis of pseudogout or gout<br>THEN a diagnostic aspiration of the painfully swollen red joint should be performed that day.	Saliba et al. (2004)	OA not related to specific sites	Nursing homes in the US
Diagnostic aspiration	IF a homebound patient has monoarticular joint pain associated with redness, warmth, or swelling AND the patient also has an oral temperature greater than 38.0 °C and does not have a previously established diagnosis of pseudogout or gout, THEN diagnostic aspiration of the painfully swollen, red joint should be performed that day.	Smith et al. (2007)	OA not related to specific sites	Home-based primary care setting in the US
Diagnostic aspiration	If a patient with knee OA has a recurrent clinically evident effusion, then he/she should be further assessed (with aspiration and analysis of synovial fluid) in order to differentiate from inflammation caused by other arthritis.	Grypdonck et al. (2014)	Knee OA	Entire spectrum of disciplines
Inventory of health-related problems	Inventory of health-related problems according to the International Classification of Functioning, Disability and Health (ICF)	Peter et al. (2013)	Knee and hip OA	Physiotherapy care in the Netherlands
Inventory of health-related problems	Assessing the presence of personal and environmental problems in so far as these relate to the limitations in activities and restrictions in participation	Peter et al. (2013)	Knee and hip OA	Physiotherapy care in the Netherlands
Inventory of health-related problems	If you have problems with daily activities, have these problems been assessed by a health professional?	Østerås et al. (2018)	OA not related to specific sites	Norwegian healthcare system
Inventory of health-related problems	Assessing the presence of hip and knee OA-specific ‘red flags’	Peter et al. (2013)	Knee and hip OA	Physiotherapy care in the Netherlands
Examination of joint before drug treatment	IF a non-OTC drug is newly prescribed to treat new joint pain THEN evidence that the affected joint was examined should be documented within 4 weeks	Saliba et al. (2004)	OA not related to specific sites	Nursing homes in the US
Examination of joint before drug treatment	IF a patient is begun on a drug treatment for joint pain, arthritis, or arthralgia, THEN evidence that the affected joint was examined should be documented.	MacLean et al. (2004)	OA not related to specific sites	US healthcare system

Evaluating treatment	Evaluating treatment with the recommended measurement instruments	Peter et al. (2013)	Knee and hip OA	Physiotherapy care in the Netherlands
Evaluating treatment	Evaluating treatment with the combination of a questionnaire and a performance test	Peter et al. (2013)	Knee and hip OA	Physiotherapy care in the Netherlands
Evaluating treatment	Evaluating treatment with a patient-specific complaint list	Peter et al. (2013)	Knee and hip OA	Physiotherapy care in the Netherlands
Evaluating treatment	Evaluating treatment with the Timed Up and Go test (TUG)	Peter et al. (2013)	Knee and hip OA	Physiotherapy care in the Netherlands
**Process: education and information**				
Information and advice	Information and advice (benchmark >90%)	Jansen et al. (2010)	Knee and hip OA	Physiotherapy care in the Netherlands.
Information concerning knowledge and understanding of OA	Providing information concerning knowledge and understanding of OA of the hip and/or knee	Peter et al. (2013)	Knee and hip OA	Physiotherapy care in the Netherlands.
Information concerning development of disease	Have you been given information about osteoarthritis from a health professional?	Østerås et al. (2018)	OA not related to specific sites	Norwegian healthcare system
Information concerning consequences for on functional performance	Providing information concerning the consequences for the patient's functional performance in terms of movements, activities and participation	Peter et al. (2013)	Knee and hip OA	Physiotherapy care in the Netherlands.
Information concerning relationship between burden and tolerance level	Providing information concerning the relationship between burden and tolerance level	Peter et al. (2013)	Knee and hip OA	Physiotherapy care in the Netherlands.
Information concerning coping style with health problems	Providing information concerning the way a patient copes with health problems	Peter et al. (2013)	Knee and hip OA	Physiotherapy care in the Netherlands.

Information concerning active and healthy lifestyle	Providing information concerning what constitutes an active and healthy lifestyle (in terms of exercise and nutrition/overweight)	Peter et al. (2013)	Knee and hip OA	Physiotherapy care in the Netherlands.
Information concerning behavioural change	Providing information concerning behavioural change (regarding physical activity)	Peter et al. (2013)	Knee and hip OA	Physiotherapy care in the Netherlands.
Information concerning joint protection and the use of aids	Providing information concerning joint protection and the use of aids	Peter et al. (2013)	Knee and hip OA	Physiotherapy care in the Netherlands.
Advice about using medications to relieve joint pain	You have been offered advice about medications (to relieve joint pain)	Blackburn et al. (2016)	OA not related to specific sites	Primary care setting in the UK
Information concerning treatment, and self-management	If a patient has knee OA, he/she should be given information access and education about the objectives of treatment and the importance of changes in lifestyle, exercise, pacing of activities, weight reduction, and other measures to unload the damaged joints.	Grypdonck et al. (2014)	Knee OA	Entire spectrum of disciplines
Information about self-management for OA	You have received advice and support on how you might help yourself to manage or deal with your joint problem	Blackburn et al. (2016)	OA not related to specific sites	Primary care setting in the UK
Information concerning pathology of OA, treatment, and self-management	The percentage of patients with symptomatic osteoarthritis, whose notes contain a record that they have been offered education regarding the natural history, treatment, and self-management of the disease at least once.	Broadbent et al. (2008)	OA not related to specific sites	Primary care in the UK general practice
Information pathology of OA, treatment, and self-management	IF an ambulatory person aged ≥ 50 years has a diagnosis of symptomatic osteoarthritis, THEN education regarding the natural history, treatment and self-management of the disease should be offered at least once.	Hardcastle et al. (2015)	OA not related to specific sites	UK healthcare system
Information concerning pathology of OA,	IF an ambulatory vulnerable elder is diagnosed with symptomatic osteoarthritis THEN education regarding the natural history, treatment, and self-management of the disease should be offered at least once within 6 months of	MacLean et al. (2001)	OA not related to specific sites	US healthcare system

treatment, and self-management	diagnosis BECAUSE such education produces improvements in physical functioning and pain.			
Information concerning pathology of OA, treatment, and self-management	IF a patient COX has had a diagnosis of symptomatic osteoarthritis for 12 months or longer THEN there should be evidence that the patient was offered education regarding the natural history, treatment, and selfmanagement of the disease at least once since the time of diagnosis BECAUSE such education produces improvements in physical functioning and pain.	MacLean et al. (2001)	OA not related to specific sites	US healthcare system
Information concerning pathology of OA, treatment, and self-management	IF a patient has had a diagnosis of symptomatic osteoarthritis of the knee or hip for >3 months, THEN education about the natural history, treatment, and self-management of osteoarthritis should have been given or recommended at least once.	MacLean et al. (2004)	knee/hip OA	US healthcare system
Information concerning pathology of OA, treatment, and self-management	IF an ambulatory person aged 65 or older has a diagnosis of symptomatic osteoarthritis, THEN education regarding the natural history, treatment and selfmanagement of the disease should be offered at least once.	Steel et al. (2004)	OA not related to specific sites	Primary and secondary care setting in the UK
Information concerning treatment options	Have you been given information about different treatment alternatives?	Østerås et al. (2018)	OA not related to specific sites	Norwegian healthcare system
Information concerning how to live with the disease	Have you been given information about how you can self-manage the disease?	Østerås et al. (2018)	OA not related to specific sites	Norwegian healthcare system
Information concerning importance of physical activity and exercise	Have you been given information about the importance of physical activity and exercise?	Østerås et al. (2018)	OA not related to specific sites	Norwegian healthcare system
Information regarding resources and tools available	Percentage of patients who receive information regarding resources and tools available for management while waiting for first musculoskeletal specialty contact.	Barber et al. (2016)	OA not related to specific sites	Centralized intake care system in Canada

for management while waiting				
**Process: exercise therapy**				
Exercise therapy for activities	Exercise therapy for activities (benchmark >90%)	Jansen et al. (2010)	Knee and hip OA	Physiotherapy care in the Netherlands
Exercise therapy for body functions	Exercise therapy for body functions (benchmark >90%)	Jansen et al. (2010)	Knee and hip OA	Physiotherapy care in the Netherlands
Walking exercises	Treating patients with walking exercises	Peter et al. (2013)	Knee and hip OA	Physiotherapy care in the Netherlands
Strengthening exercises	Treating patients with strengthening of muscles.	Peter et al. (2013)	Knee and hip OA	Physiotherapy care in the Netherlands
Strengthening or aerobic exercises	IF an ambulatory person aged ≥ 50 years has had a diagnosis of symptomatic osteoarthritis of the knee for longer than 3 months and has no contraindications to exercise and is physically and mentally able to exercise, THEN a directed or supervised strengthening or aerobic exercise programme should have been prescribed at least once.	Hardcastle et al. (2015)	OA not related to specific sites	UK healthcare system
Strengthening or aerobic exercises	IF an ambulatory vulnerable elder is newly diagnosed with osteoarthritis of the knee, has no contraindication to exercise, and is physically and mentally able to exercise, THEN a directed or supervised strengthening or aerobic exercise program should be prescribed within 3 months of diagnosis BECAUSE such programs improve functional status and reduce pain.	MacLean et al. (2001)	Knee OA	US healthcare system
Strengthening or aerobic exercises	IF an ambulatory vulnerable elder has had a diagnosis of symptomatic osteoarthritis of the knee for longer than 12 months, has no contraindication to exercise, and is physically and mentally able to exercise, THEN there should be evidence that a directed or supervised strengthening or aerobic exercise program was prescribed at least once since the time of diagnosis BECAUSE such programs improve functional status and reduce pain.	MacLean et al. (2001)	Knee OA	US healthcare system
Strengthening or aerobic exercises	IF an ambulatory VE has symptomatic OA of the knee or hip for longer than 3 months and is able to exercise, THEN a	MacLean et al. (2007)	Knee and hip OA	US healthcare system

	directed or supervised muscle strengthening or aerobic exercise program should be recommended and activity reviewed annually, BECAUSE directed or supervised exercise programs improve functional status and reduce pain.			
Strengthening or aerobic exercises	IF an ambulatory patient has had a diagnosis of symptomatic osteoarthritis of the knee or hip for >3 months AND has no contraindication to exercise and is physically and mentally able to exercise, THEN a directed or supervised muscle strengthening or aerobic exercise program should have been prescribed at least once and reviewed at least once per year.	MacLean et al. (2004)	Knee/hip OA	US healthcare system
Strengthening or aerobic exercises	IF an ambulatory NH resident is newly diagnosed with symptomatic osteoarthritis of the knee and has no contraindication to exercise and is physically and mentally able to exercise THEN a directed or supervised strengthening or aerobic exercise program should be prescribed within 1 month of diagnosis	Saliba et al. (2004)	Knee OA	Nursing homes in the US
Strengthening or aerobic exercises	IF an ambulatory NH resident has a diagnosis of symptomatic knee osteoarthritis for >3 months, has no contraindication to exercise, and is physically and mentally able to exercise THEN there should be evidence that a directed or supervised strengthening or aerobic exercise program was prescribed at least once since the time of diagnosis	Saliba et al. (2004)	Knee OA	Nursing homes in the US
Strengthening or aerobic exercises	IF an ambulatory homebound patient is newly diagnosed with osteoarthritis of the knee, has no contraindication to exercise, and is physically and mentally able to exercise, THEN a directed or supervised strengthening or aerobic exercise program should be prescribed within 3 months of diagnosis.	Smith et al. (2007)	Knee OA	Home-based primary care setting in the US
Strengthening or aerobic exercises	IF an ambulatory person aged 65 or older has had a diagnosis of symptomatic osteoarthritis of the knee for longer than 3 months and has no contraindications to exercise and is physically and mentally able to exercise, THEN a directed or supervised strengthening or aerobic	Steel et al. (2004)	Knee OA	Primary and secondary care setting in the UK

	exercise programme should have been prescribed at least once.			
Strengthening or aerobic exercises	IF an ambulatory homebound patient has had a diagnosis of symptomatic osteoarthritis of the knee for longer than 12 months and is physically and mentally able to exercise, THEN there should be evidence that a physical therapy evaluation for focused strengthening exercises was prescribed at least once since the time of diagnosis.	Smith et al. (2007)	Knee OA	Home-based primary care setting in the US
Strengthening or aerobic exercises	(Patients with knee/hip OA who have documented recommendations for general aerobic and/or muscle strengthening exercise at least once per year, unless contraindicated (e.g. significant heart failure)/ Total number of patients with KHOA without contraindications for general aerobic exercise) * 100: the higher, the better the quality of care	Doubova et al. (2015)	Knee/hip OA	Primary care setting; family medicine in Mexico
Strengthening, aerobic, and functional exercises	If a patient has knee OA, then exercise therapy should be prescribed, including at least muscle strengthening, aerobic exercises and functional exercises, and combined with range of motion exercises in case of range of motion restrictions.	Grypdonck et al. (2014)	Knee OA	Entire spectrum of disciplines
Improving aerobic capacity	Treating patients with improving of aerobic capacity	Peter et al. (2013)	Knee and hip OA	Physiotherapy care in the Netherlands
Functional exercises	Treating patients with functional exercises	Peter et al. (2013)	Knee and hip OA	Physiotherapy care in the Netherlands
Exercise therapy	Providers caring for patients with symptoms of hip or knee OA should recommend both of the following at least once in 2 years: exercise programs for persons with hip or knee OA.	Moore et al. (2000)	OA not related to specific sites	US healthcare system
Exercise therapy	Providers caring for patients with symptoms of hip or knee osteoarthritis should recommend exercise programs at least once in 2 years.	Asch et al. (2004)	Knee/hip OA	US healthcare system
Advice on exercise or activities	You have been offered information or advice on exercise or activity to help with your joint problem	Blackburn et al. (2016)	OA not related to specific sites	Primary care setting in the UK

Exercise therapy tailored to patients' goals	If a patient with knee OA is following exercise therapy, then the content and intensity of the exercise program should be tailored to the patient's individual goals in terms of limitations of activity and restrictions of participation.	Grypdonck et al. (2014)	Knee OA	Entire spectrum of disciplines
Frequency of exercise therapy sessions	If a patient with knee OA is following exercise therapy, then the treatment sessions should be spread over longer periods with lower frequencies in the later stages of the exercise program to facilitate the transition from exercise therapy to independent exercising and maintaining sufficient level of physical activity.	Grypdonck et al. (2014)	Knee OA	Entire spectrum of disciplines
Regular evaluations of exercise therapy	If a patient with knee OA is following exercise therapy, then regular evaluations by the physiotherapist are necessary. To make the switchover from a supervised to an autonomous program, an evaluation session should be performed every 3 months in the first year, every 6 months in the second year, and once per year afterward.	Grypdonck et al. (2014)	Knee OA	Entire spectrum of disciplines
Combining exercise therapy with education/self-management interventions	If a patient with knee OA is following exercise therapy, then the exercise therapy should be combined with education/self-management interventions to improve patients' mental and physical performance and to alleviate pain.	Grypdonck et al. (2014)	Knee OA	Entire spectrum of disciplines
**Process: weight counselling**				
Advice about body weight and joint pain	You have received advice about body weight and joint pain	Blackburn et al. (2016)	OA not related to specific sites	Primary care setting in the UK
Advice to lose weight	(Overweight (BMI ≥27 kg/m2) patients with KHOA who have documented nutritional counselling provided by the Nutrition and Dietary Service and/or who were encouraged by their family physician at least one time per year to lose weight/ Total number of overweight patients with KHOA) * 100: the higher, the better the quality of care	Doubova et al. (2015)	Knee/hip OA	Primary care setting; family medicine in Mexico
Advice to lose weight	If a patient with knee OA is overweight, then he/she should be encouraged to lose weight and maintain his/her weight at a lower level.	Grypdonck et al. (2014)	Knee OA	Entire spectrum of disciplines

Advice to lose weight	IF an individual is overweight (as defined by body mass index of >27 kg/m2), THEN the individual should be advised to lose weight annually.	MacLean et al. (2004)	OA not related to specific sites	US healthcare system
Advice to lose weight	IF a patient has symptomatic osteoarthritis of the knee or hip and is overweight (as defined by body mass index of >27 kg/m2), THEN the patient should be advised to lose weight at least annually AND the benefit of weight loss on the symptoms of osteoarthritis should be explained to the patient.	MacLean et al. (2004)	Knee/hip OA	US healthcare system
Advice to lose weight	Providers caring for patients with symptoms of hip or knee OA should recommend weight loss among persons with knee OA and a BMI >25 following at least once in 2 years.	Moore et al. (2000)	OA not related to specific sites	US healthcare system
Advice to lose weight	Have you been advised to lose weight, if you are overweight?	Østerås et al. (2018)	OA not related to specific sites	Norwegian healthcare system
**Process: 'do not do' QIs**				
No massage therapy	No massage therapy (benchmark <10%)	Jansen et al. (2010)	Knee and hip OA	Physiotherapy care in the Netherlands
No physical modalities	No use of physical modalities other than TENS (e.g. pulsed shortwave) (benchmark <10%)	Jansen et al. (2010)	Knee and hip OA	Physiotherapy care in the Netherlands
No brace prescription	If a patient has knee OA, then a brace should not be prescribed (except in unicompartmental knee OA with axial deviation).	Grypdonck et al. (2014)	Knee OA	Entire spectrum of disciplines
**Process: pharmacological treatment**				
Paracetamol as first-line pharmacologic therapy	Patients who were prescribed paracetamol (numerator)/ all patients with a drug prescription for osteoarthritis in the past month (denominator)	VandenBerghe et al. (2004)	OA not related to specific sites	General practice in Belgium (primary care)
Paracetamol as first-line pharmacologic therapy	Patients with a new diagnosis of osteoarthritis who wish to take medication for joint symptoms should be offered a trial of paracetamol if not already tried.	Marshall et al. (2003)	OA not related to specific sites	General practices in the UK health care systems
Paracetamol as first-line pharmacologic therapy	Patients with a new diagnosis of OA who wish to take medication for joint symptoms should be offered a trial of acetaminophen.	Moore et al. (2000)	OA not related to specific sites	US healthcare system

Paracetamol as first-line pharmacologic therapy	IF oral pharmacological therapy is initiated to treat osteoarthritis among people aged ≥ 50 years, THEN paracetamol should be the first drug used, unless there is a contraindication to use.	Hardcastle et al. (2015)	OA not related to specific sites	UK healthcare system
Paracetamol as first-line pharmacologic therapy	Patients with a new diagnosis of osteoarthritis who wish to take medication for joint symptoms should be offered a trial of acetaminophen.	Asch et al. (2004)	OA not related to specific sites	US healthcare system
Paracetamol as first-line pharmacologic therapy	The percentage of patients in whom oral pharmacological therapy was initiated to treat osteoarthritis, whose notes contain a record that they were offered paracetamol first (unless contraindicated)	Broadbent et al. (2008)	OA not related to specific sites	Primary care in the UK general practice
Paracetamol as first-line pharmacologic therapy	IF oral pharmacologic therapy is initiated to treat osteoarthritis in a vulnerable elder, THEN acetaminophen should be the first drug used, unless there is a documented contraindication to use, BECAUSE this agent is as effective in treating osteoarthritis as other oral agents, and it is less toxic.	MacLean et al. (2001)	OA not related to specific sites	US healthcare system
Paracetamol as first-line pharmacologic therapy	IF a VE is started on pharmacological therapy to treat OA, THEN acetaminophen should be tried first, BECAUSE acetaminophen achieves pain relief comparable to that of an NSAID (nonselective and selective) for many patients and is associated with a lower burden of common serious adverse events.	MacLean et al. (2007)	OA not related to specific sites	US healthcare system
Paracetamol as first-line pharmacologic therapy	IF a nonnarcotic pharmacologic therapy is initiated to treat osteoarthritis pain of mild or moderate severity, THEN acetaminophen should be the first drug used, unless there is a documented contraindication to use.	MacLean et al. (2004)	OA not related to specific sites	US healthcare system
Paracetamol as first-line pharmacologic therapy	IF oral pharmacologic therapy is initiated to treat osteoarthritis THEN acetaminophen should be the first drug used	Saliba et al. (2004)	OA not related to specific sites	Nursing homes in the US
Paracetamol as first-line pharmacologic therapy	IF oral pharmacologic therapy is initiated to treat osteoarthritis in a homebound patient, THEN acetaminophen should be the first drug used, unless there is a documented contraindication to use	Smith et al. (2007)	OA not related to specific sites	Home-based primary care setting in the US

Paracetamol as first-line pharmacologic therapy	IF oral pharmacological therapy is initiated to treat osteoarthritis among people aged 65 or older, THEN paracetamol should be the first drug used, unless there is a contraindication to use.	Steel et al. (2004)	OA not related to specific sites	Primary and secondary care setting in the UK
Paracetamol as first-line pharmacologic therapy	IF oral pharmacological therapy is initiated to treat osteoarthritis in an elder, THEN paracetamol (acetaminophen) should be the first drug used, UNLESS there is a documented contra-indication.	Wierenga et al. (2011)	OA not related to specific sites	Dutch in-hospital pharmaceutical care
Paracetamol as first-line pharmacologic therapy	If you have joint pain, was paracetamol the first medication that was recommended?	Østerås et al. (2018)	OA not related to specific sites	Norwegian healthcare system
Paracetamol as first-line pharmacologic therapy	(Patients with newly diagnosed of KHOA who received prescription of acetaminophen as initial oral analgesic, otherwise contraindicated/ Total number of patients with recent diagnosis of KHOA) * 100: the higher, the better the quality of care	Doubova et al. (2015)	Knee/hip OA	Primary care setting; family medicine in Mexico
Paracetamol as first-line pharmacologic therapy	If a patient has knee OA, then acetaminophen up to 3 g/day should be used as the initial oral analgesic.	Grypdonck et al. (2014)	Knee OA	Entire spectrum of disciplines
Trial of maximum-dose acetaminophen before changing from acetaminophen to different oral agent	The percentage of patients in whom oral pharmacological therapy was changed from paracetamol to a different oral agent, whose notes contain a record that they were offered a trial of maximum-dose paracetamol	Broadbent et al. (2008)	OA not related to specific sites	Primary care in the UK general practice
Trial of maximum-dose acetaminophen before changing from acetaminophen to different oral agent	IF oral pharmacologic therapy for osteoarthritis in a vulnerable elder is changed from acetaminophen to a different oral agent, THEN there should be evidence that the patient has had a trial of maximum-dose acetaminophen (suitable for age and comorbid conditions) BECAUSE acetaminophen, in adequate doses, is as effective in treating osteoarthritis as other oral agents, and it is less toxic.	MacLean et al. (2001)	OA not related to specific sites	US healthcare system

Trial of maximum-dose acetaminophen before changing from acetaminophen to different oral agent	IF oral pharmacologic therapy for osteoarthritis is changed from acetaminophen to a different oral agent, THEN there should be evidence that the patient has had a trial of maximum-dose acetaminophen (suitable for age/comorbidities).	MacLean et al. (2004)	OA not related to specific sites	US healthcare system
Trial of maximum-dose acetaminophen before changing from acetaminophen to different oral agent	IF oral pharmacologic therapy for symptomatic osteoarthritis is changed from acetaminophen to a different oral agent THEN there should be evidence that the NH resident has had a trial of maximum dose acetaminophen (suitable for age and comorbid conditions)	Saliba et al. (2004)	OA not related to specific sites	Nursing homes in the US
Trial of maximum-dose acetaminophen before changing from acetaminophen to different oral agent	IF oral pharmacologic therapy for osteoarthritis in a homebound patient is changed from acetaminophen to a different oral agent, THEN there should be evidence that the patient has had a trial of maximum-dose acetaminophen (suitable for age and comorbid conditions).	Smith et al. (2007)	OA not related to specific sites	Home-based primary care setting in the US
Trial of maximum-dose acetaminophen before changing from acetaminophen to different oral agent	IF oral pharmacological therapy for osteoarthritis is changed from paracetamol to a different oral agent among people aged 65 or older, THEN the patient should have had a trial of maximum dose paracetamol (suitable for age/co-morbidities).	Steel et al. (2004)	OA not related to specific sites	Primary and secondary care setting in the UK
Trial of maximum-dose acetaminophen before changing from	IF oral pharmacological therapy for osteoarthritis in an elder is changed from paracetamol (acetaminophen) to a different oral agent, THEN there should be evidence that the patient has had a trial of maximum dose of paracetamol (suitable for age and co-morbid conditions.	Wierenga et al. (2011)	OA not related to specific sites	Dutch in-hospital pharmaceutical care

acetaminophen to different oral agent				
NSAID prescription	Patients which were not prescribed an NSAID (numerator)/ all patients with a drug prescription for osteoarthritis in the past month (denominator)	VandenBerghe et al. (2004)	OA not related to specific sites	General practice in Belgium (primary care)
NSAID prescription	Patients who were prescribed a coxib (numerator)/ all patients who received an NSAID for osteoarthritis in the past month (denominator)	VandenBerghe et al. (2004)	OA not related to specific sites	General practice in Belgium (primary care)
NSAID prescription	Patients who did not received a repeated prescription/ all patients who received an NSAID for osteoarthritis in the past month (denominator)	VandenBerghe et al. (2004)	OA not related to specific sites	General practice in Belgium (primary care)
NSAID prescription	If NSAIDs are considered, Ibuprofen should be considered for first line treatment unless contraindicated or intolerant.	Marshall et al. (2003)	OA not related to specific sites	General practices in the UK health care systems.
NSAID prescription	The percentage of patients with osteoarthritis treated with an NSAID, whose notes contain a record that ibuprofen (or a cox-2 inhibitor) has been considered for first-line treatment (unless contraindicated or intolerant)	Broadbent et al. (2008)	OA not related to specific sites	Primary care in the UK general practice
NSAID prescription	(Patients aged 65 years or older with KHOA and one of the following comorbidities (history of peptic ulcer disease or gastrointestinal bleeding, chronic kidney disease, cardiac insufficiency and/or those receiving anticoagulant or glucocorticoids) who receive NSAID prescription/ Total number of patients aged 65 years or older with KHOA and one of the previously mentioned comorbidities)* 100= the lower, the better the quality of care	Doubova et al. (2015)	Knee/hip OA	Primary care setting; family medicine in Mexico
NSAID prescription	If a patient has knee OA and there is no adequate response on acetaminophen, or there is severe pain and/or inflammation, then oral NSAID should be used.	Grypdonck et al. (2014)	Knee OA	Entire spectrum of disciplines
NSAID prescription	If NSAID are used in a patient with knee OA, then they should be used intermittently (max 3 weeks sustained use) and at the lowest effective dose.	Grypdonck et al. (2014)	Knee OA	Entire spectrum of disciplines
NSAID prescription	If a patient with knee OA has heart failure grade 2–4, ischemic heart disease, or renal insufficiency with a GFR < 40	Grypdonck et al. (2014)	Knee OA	Entire spectrum of disciplines

BMJ Publishing Group Limited (BMJ) disclaims all liability and responsibility arising from any reliance placed on this supplemental material which has been supplied by the author(s)

	ml/min, then NSAID should not be used. In case of other cardiovascular risk factors (e.g., hypertension, …), NSAID should be used with caution.			
NSAID prescription concomitant with either misoprostol or proton-pump inhibitor	Patients with osteoarthritis prescribed oral NSAIDs who are at high risk of gastrointestinal side effects (past history of dyspepsia or known peptic ulcer) should be considered for a co-prescription of PPIs, H2 antagonists or Misoprolol, unless contraindicated or intolerant	Marshall et al. (2003)	OA not related to specific sites	General practices in the UK health care systems.
NSAID prescription concomitant with either misoprostol or proton-pump inhibitor	(Patients with KHOA and high risk for gastrointestinal complications who received NSAID prescription concomitant with either misoprostol or a proton-pump inhibitor/ Total number of patients with KHOA and high risk of gastrointestinal complications who received NSAIDs) * 100= the higher, the better the quality of care.	Doubova et al. (2015)	Knee/hip OA	Primary care setting; family medicine in Mexico
NSAID prescription concomitant with either misoprostol or proton-pump inhibitor	If a patient with knee OA and a history of bleeding gastric ulcers has a need for NSAID, then either a COX-2 selective agent or a non-selective NSAID with coprescription of a proton pump inhibitor/misoprostol should be used instead of a non-selective NSAID.	Grypdonck et al. (2014)	Knee OA	Entire spectrum of disciplines
NSAID prescription concomitant with either misoprostol or proton-pump inhibitor	IF a VE with a risk factor for GI bleeding (aged ≥75, peptic ulcer disease, history of GI bleeding, warfarin use, chronic glucocorticoid use) is treated with a nonselective NSAID, THEN he or she should be treated concomitantly with misoprostol or a proton pump inhibitor (PPI)	MacLean et al. (2007)	OA not related to specific sites	US healthcare system
NSAID prescription concomitant with either misoprostol or proton-pump inhibitor	IF a VE with two or more risk factors for GI bleeding (aged ≥75, peptic ulcer disease, history of GI bleeding, warfarin use, chronic glucocorticoid use) is treated with daily aspirin, THEN he or she should be treated concomitantly with either misoprostol or a PPI, BECAUSE this will reduce the risk of GI bleeding.	MacLean et al. (2007)	OA not related to specific sites	US healthcare system
NSAID prescription concomitant with either misoprostol	IF a vulnerable elder is older than 75 years of age, is treated with warfarin, or has a history of peptic ulcer disease or gastrointestinal bleeding, AND is being treated with a COX	MacLean et al. (2001)	OA not related to specific sites	US healthcare system

or proton-pump inhibitor	nonselective NSAID, THEN he or she should be offered concomitant treatment with either misoprostol or a proton-pump inhibitor BECAUSE this will substantially reduce the risk for NSAID-induced gastrointestinal bleeding.			
Informing patients about risks of medication use	IF a patient is treated with a COX nonselective nonsteroidal anti-inflammatory drug (NSAID), THEN there should be evidence that the patient was advised of the risk for gastrointestinal bleeding associated with these drugs BECAUSE this risk is substantial.	MacLean et al. (2001)	OA not related to specific sites	US healthcare system
Informing patients about risks of medication use	IF a patient is treated with a COX-nonselective NSAID, THEN there should be evidence that the patient was advised of the risk for gastrointestinal bleeding, as well as cardiovascular risk associated with these drugs.	Smith et al. (2007)	OA not related to specific sites	Home-based primary care setting in the US
Informing patients about risks of medication use	The percentage of patients with osteoarthritis treated with an NSAID, whose notes contain a record that they have been advised of the gastrointestinal and renal risks associated with this drug	Broadbent et al. (2008)	OA not related to specific sites	Primary care in the UK general practice
Screening patients for risks of medication use	The percentage of patients with osteoarthritis regularly treated with an NSAID, whose notes contain a record that they have been asked about gastrointestinal symptoms within the previous 12 months	Broadbent et al. (2008)	OA not related to specific sites	Primary care in the UK general practice
Informing patients about risks of medication use	If you use anti-inflammatory medications, have you been given information about the effects and possible side-effects of this medication? (e.g. ibuprofen (Nurofen, Brufen), diclofenac (Voltarol), naproxen (Naprosyn),celecoxib (Celebrex))	Østerås et al. (2018)	OA not related to specific sites	Norwegian healthcare system
Informing patients about risks of medication use	IF aVE is prescribed chronic high-dose acetaminophen (≥3 g/d) or a VE with liver disease is prescribed chronic acetaminophen, THEN he or she should be advised of the risk of liver toxicity, BECAUSE these risks are greater with high doses of acetaminophen and when underlying liver disease is present.	MacLean et al. (2007)	OA not related to specific sites	US healthcare system

Informing patients about risks of medication use	IF a VE is prescribed an NSAID (non- selective or selective), THEN GI bleeding risks should be discussed and documented	MacLean et al. (2007)	OA not related to specific sites	US healthcare system
Informing patients about risks of medication use	IF a VE is prescribed daily aspirin (including low-dose,<325mg/d), THEN GI bleeding risks should be discussed and documented, BECAUSE selective NSAIDs, non-selective NSAIDs, and aspirin increase the risk of bleeding.	MacLean et al. (2007)	OA not related to specific sites	US healthcare system
Corticosteroid injection	If you have experienced an acute deterioration of your symptoms, have you been given or offered a steroid injection?	Østerås et al. (2018)	OA not related to specific sites	Norwegian healthcare system
Drug prescription	Patients without a drug prescription for osteoarthritis in the past month (numerator)/ all patients with osteoarthritis (denominator)	VandenBerghe et al. (2004)	OA not related to specific sites	General practice in Belgium (primary care)
Chondroitin and glucosamine-chondroitin	If a patient has knee OA, then chondroitin and glucosamine-chondroitin combination products should not be used.	Grypdonck et al. (2014)	Knee OA	Entire spectrum of disciplines
Opioids	If a patient has knee OA, then strong opioids (oxymorphone, oxycodone, fentanyl, morphine sulfate) should not be used.	Grypdonck et al. (2014)	Knee OA	Entire spectrum of disciplines
Offering stronger pain killers (e.g., co-proxamol, co-dydramol, tramadol, co-codamol, dihydrocodeine, codeine)	If you have prolonged severe joint pain, which is not relieved sufficiently by paracetamol, have you been offered stronger pain killing medications? (e.g. co-codamol, codeine, tramadol, co-proxamol, co-dydramol, dihydrocodeine)	Østerås et al. (2018)	OA not related to specific sites	Norwegian healthcare system
**Process: referral**				
Referral to exercise or activity programmes	You have been offered a referral to an exercise or activity programme for your joint problem.	Blackburn et al. (2016)	OA not related to specific sites	Primary care setting in the UK
Referral to physiotherapy	You have been offered a referral for physiotherapy for your joint problem.	Blackburn et al. (2016)	OA not related to specific sites	Primary care setting in the UK
Referral to a physical therapist	If a patient has symptomatic knee OA, then he/she has to be referred to a physical therapist for instruction of the patient	Grypdonck et al. (2014)	Knee OA	Entire spectrum of disciplines

	in appropriate exercises, for motivation of the patient to implement exercise and adhere to exercise, and to evaluate performance.			
Referral for physical activity and exercise therapy	Have you been referred or offered a referral to a health professional who can advise you about physical activity and exercise?	Østerås et al. (2018)	OA not related to specific sites	Norwegian healthcare system
Referral to regular community exercise and sports activities after a period of supervised exercise.	If a patient with knee OA is following exercise therapy, then he/she should be referred to regular community exercise and sports activities after a period of supervised exercise.	Grypdonck et al. (2014)	Knee OA	Entire spectrum of disciplines
Referral to weight loss services	You have received a referral for weight loss services.	Blackburn et al. (2016)	OA not related to specific sites	Primary care setting in the UK
Referral to weight loss services	IF a patient has symptomatic osteoarthritis of the knee or hip and has been overweight (as defined by body mass index of ≥27 kg/m2 for >3 years), THEN the patient should receive referral to a weight loss program.	MacLean et al. (2004)	Hip/knee OA	US healthcare system
Referral to weight loss services	Have you been referred or offered a referral to someone who can help you to lose weight, if you are overweight?	Østerås et al. (2018)	OA not related to specific sites	Norwegian healthcare system
Referral to an orthopaedic surgeon	IF a vulnerable elder with severe symptomatic osteoarthritis of the knee or hip has failed to respond to nonpharmacologic and pharmacologic therapy and has no contraindication to surgery, THEN the patient should be referred to an orthopedic surgeon to be evaluated for total joint replacement within 6 months unless a contraindication to surgery is documented BECAUSE hip and knee replacements markedly improve function and quality of life by reducing pain and/or improving range of motion.	MacLean et al. (2001)	Knee/hip OA	US healthcare system
Referral to an orthopaedic surgeon	IF a VE has severe symptomatic OA of the knee or hip despite nonsurgical therapy, THEN a referral to an orthopedic surgeon should be made, BECAUSE joint surgery	MacLean et al. (2007)	Hip/knee OA	US healthcare system

	may reduce pain and improve functional status and quality of life.			
Referral to an orthopaedic surgeon	Patients with severe symptomatic osteoarthritis of the knee or hip who have failed to respond to conservative therapy should be offered referral to an orthopaedic surgeon for consideration of joint replacement.	Marshall et al. (2003)	OA not related to specific sites	General practices in the UK health care systems.
Referral to an orthopaedic surgeon	IF a patient with severe symptomatic osteoarthritis of the knee or hip has failed to respond to nonpharmacologic and pharmacologic therapy, THEN the patient should be offered referral to an orthopedic surgeon.	MacLean et al. (2004)	Hip/knee OA	US healthcare system
Referral to an orthopaedic surgeon	IF a person aged ≥ 50 years with severe symptomatic osteoarthritis of the knee or hip has failed to respond to non-pharmacological and pharmacological therapy, THEN the patient should be offered referral to an orthopaedic surgeon to be evaluated for total joint replacement within 6 months unless surgery is contraindicated.	Hardcastle et al. (2015)	OA not related to specific sites	UK healthcare system
Referral to an orthopaedic surgeon	IF a person aged 65 or older with severe symptomatic osteoarthritis of the knee or hip has failed to respond to non- pharmacological and pharmacological therapy, THEN the patient should be offered referral to an orthopaedic surgeon to be evaluated for total joint replacement within 6 months unless surgery is contraindicated.	Steel et al. (2004)	Hip/knee OA	Primary and secondary care setting in the UK
Referral to an orthopaedic surgeon	The percentage of patients with severe symptomatic osteoarthritis of the knee or hip that has failed to respond to non-pharmacological and pharmacological therapy, whose notes contain a record that they were offered referral to an orthopaedic surgeon to be evaluated for total joint replacement within 6 months unless surgery is contraindicated.	Broadbent et al. (2008)	Hip/knee OA	Primary care in the UK general practice
Referral to an orthopaedic surgeon	If you are severely troubled by your osteoarthritis, and exercise and medication do not help, have you been referred or offered a referral for an assessment for operation? (e.g. joint replacement)	Østerås et al. (2018)	OA not related to specific sites	Norwegian healthcare system

Referral for laboratory tests	(Patients with KHOA and with NSAID prescription for 6 months or longer who were referred for the following laboratory tests (blood count, serum creatinine and liver enzymes) at least once in the previous 12 months / Total number of patients with KHOA and with NSAID prescription for 6 months or longer) * 100: the higher, the better the quality of care.	Doubova et al. (2015)	Knee/hip OA	Primary care setting; family medicine in Mexico
Referral time	Time from OA referral receipt to referral completion for initially incomplete referrals.	Barber et al. (2016)	OA not related to specific sites	Centralized intake care system in Canada
Referral time	Time from receipt of complete OA referral to musculoskeletal appointment.	Barber et al. (2016)	OA not related to specific sites	Centralized intake care system in Canada
QI specifically regarding Western Canada Waiting List	Distribution of OA referrals in each urgency category (as scored using the Western Canada Waiting List referral tool).	Barber et al. (2016)	OA not related to specific sites	Centralized intake care system in Canada
QI specifically regarding Western Canada Waiting List	Percentage of OA referrals triaged as highest urgency based on high Western Canada Waiting List priority criteria scores seen within Wait Time Alliance benchmarks .	Barber et al. (2016)	OA not related to specific sites	Centralized intake care system in Canada
QI specifically regarding Western Canada Waiting List	Percentage of OA referrals scored using Western Canada Waiting List priority referral criteria.	Barber et al. (2016)	OA not related to specific sites	Centralized intake care system in Canada
Referrals to centralized intake care rejected or redirected	Percentage of referrals rejected or redirected when received at centralized intake.	Barber et al. (2016)	OA not related to specific sites	Centralized intake care system in Canada
Referral with complete information	Percentage of OA referrals received with complete information.	Barber et al. (2016)	OA not related to specific sites	Centralized intake care system in Canada
Referral received through centralized intake care	Number of referrals received through centralized intake	Barber et al. (2016)	OA not related to specific sites	Centralized intake care system in Canada

Agreement of severe OA in candidates for joint replacement	Agreement of centralized intake suspected diagnosis of severe OA cases (e.g., patients who are candidates for hip or knee joint replacements) versus confirmed diagnosis of severe OA.	Barber et al. (2016)	OA not related to specific sites	Centralized intake care in Canada
**Process: indication for surgery**				
Indication for knee replacement	If a patient with knee OA is not obtaining adequate pain relief and functional improvement, then he/she should be considered for joint replacement.	Grypdonck et al. (2014)	Knee OA	Entire spectrum of disciplines
Indication for unicompartmental knee replacement	If a patient has unicompartmental knee OA, then a unicompartmental knee replacement should be considered.	Grypdonck et al. (2014)	Knee OA	Entire spectrum of disciplines
No arthroscopic interventions	If a patient has knee OA, then arthroscopic interventions are not recommended. Coexisting meniscal lesions should not be treated. Only in case of locking of the knee from a large meniscal fragment or a loose body or an extension loss from an anterior anvil osteophyte is arthroscopic treatment indicated.	Grypdonck et al. (2014)	Knee OA	Entire spectrum of disciplines
Operating room time	Operating room time for arthroplasty surgeons in Alberta.	Barber et al. (2016)	OA not related to specific sites	Centralized intake care system in Canada
**Process: documentation**				
Documentation of symptoms, limitations in daily activities, systemic or inflammatory disease, and use and effectiveness of treatment ≥once in 2 years	Providers caring for patients with symptoms of OA should document all of the following at least once in 2 years:<br>a. the location of symptoms;<br>b. the presence or absence of limitations in daily activities;<br>c. the presence or absence of a history or symptoms of systemic or inflammatory disease;<br>d. the use and effectiveness of treatment modalities.	Moore et al. (2000)	OA not related to specific sites	US healthcare system
Documentation of symptoms, limitations in daily	Providers caring for patients with symptoms of osteoarthritis should document all of the following at least once in 2 years: the location of symptoms and/or the presence or absence of limitations in daily activities.	Asch et al. (2004)	OA not related to specific sites	US healthcare system

activities ≥once in 2 years				
Documentation of parameters from physical examination ≥once in 2 years	Providers caring for patients with symptoms of OA should document the following for any one affected joint at least once in 2 years:<br>a. the presence or absence of effusion;<br>b. the presence or absence of bony enlargement;<br>c. the presence or absence of tenderness;<br>d. the presence or absence of limitations in range of motion.	Moore et al. (2000)	OA not related to specific sites	US healthcare system
Documentation the presence or absence of systemic or inflammatory disease, and joint trauma or surgery in patients with incident OA	Providers caring for patients with incident symptoms of OA should document at least one of the following:<br>• the presence or absence of a history of any systemic or inflammatory disease that may mimic OA;<br>• the presence or absence of any current symptoms of systemic or inflammatory disease that may mimic OA;<br>• the presence or absence of a history of joint trauma or surgery.	Moore et al. (2000)	OA not related to specific sites	US healthcare system
Recording of problem areas	Problem areas recorded (i.e. inflammation, pain, impairments of function, activity limitations, participation restrictions, and passive coping behaviour) (benchmark >90%)	Jansen et al. (2010)	Knee and hip OA	Physiotherapy care in the Netherlands
Recording of patient profile	Patient profile recording according to the Dutch physiotherapy guidelines (benchmark >90%)	Jansen et al. (2010)	Knee and hip OA	Physiotherapy care in the Netherlands
**Process: Follow up, treatment frequency, duration and aftercare**				
Follow up review	You have been given a follow-up review of your joint problem.	Blackburn et al. (2016)	OA not related to specific sites	Primary care setting in the UK
Follow up review	Patients receiving care for symptoms of OA should be seen in follow-up at least every 6 months.	Moore et al. (2000)	OA not related to specific sites	US healthcare system
Total number of sessions	Number of sessions lower than 12	Jansen et al. (2010)	Knee and hip OA	Physiotherapy care in the Netherlands
Duration of treatment episode	Duration of treatment episode less than 6 weeks	Jansen et al. (2010)	Knee and hip OA	Physiotherapy care in the Netherlands

Treatment frequency	Treatment frequency	Jansen et al. (2010)	Knee and hip OA	Physiotherapy care in the Netherlands
Aftercare	Aftercare (e.g. home exercise programme, follow up consultation, advice to participate in community based or sport programmes)	Jansen et al. (2010)	Knee and hip OA	Physiotherapy care in the Netherlands
**Outcome**				
Healthcare providers' experience	Musculoskeletal specialty care provider experience with centralized intake.	Barber et al. (2016)	OA not related to specific sites	Centralized intake care system in Canada
Healthcare providers' experience	Administrative staff and allied health professional experience with centralized intake.	Barber et al. (2016)	OA not related to specific sites	Centralized intake care system in Canada
Healthcare providers' experience	Referring clinician's experience with centralized intake.	Barber et al. (2016)	OA not related to specific sites	Centralized intake care system in Canada
Patient experience	Patient experience with centralized intake.	Barber et al. (2016)	OA not related to specific sites	Centralized intake care system in Canada
Patient satisfaction	You are satisfied with the overall quality of the consultation with his/her GP for OA.	Blackburn et al. (2016)	OA not related to specific sites	Primary care setting in the UK
Patient satisfaction	Patients satisfaction with treatment*	Jansen et al. (2010)	Knee and hip OA	Physiotherapy care in the Netherlands
Level of pain	VAS for severity of pain decrease of more than 25%	Jansen et al. (2010)	Knee and hip OA	Physiotherapy care in the Netherlands
Level of pain and function	Global perceived effect either for pain or for restrictions in daily activities (5 point Likert scale)*	Jansen et al. (2010)	Knee and hip OA	Physiotherapy care in the Netherlands
Functional capacity	Algofunctional index decrease of more than 25%	Jansen et al. (2010)	Knee and hip OA	Physiotherapy care in the Netherlands
Achievement of treatment goals	The extent to which the treatment goals were achieved*	Jansen et al. (2010)	Knee and hip OA	Physiotherapy care in the Netherlands

* No specific threshold reported in the article; QIs are developed based on the Dutch physiotherapy guideline and further information on thresholds is documented in the recommendations of this guideline.
